# Song recordings suggest feeding ground sharing in Southern Hemisphere humpback whales

**DOI:** 10.1038/s41598-022-17999-y

**Published:** 2022-08-17

**Authors:** Elena Schall, Divna Djokic, Erin C. Ross-Marsh, Javier Oña, Judith Denkinger, Julio Ernesto Baumgarten, Linilson Rodrigues Padovese, Marcos R. Rossi-Santos, Maria Isabel Carvalho Gonçalves, Renata Sousa-Lima, Rodrigo Hucke-Gaete, Simon Elwen, Susannah Buchan, Tess Gridley, Ilse Van Opzeeland

**Affiliations:** 1 Ocean Acoustics Group, Alfred Wegener Institute Helmholtz Centre for Polar and Marine Research, Klußmannstraße 3d, 27570 Bremerhaven, Germany; 2 Laboratory of Bioacoustics, Federal University of Rio Grande do Norte, Natal, Brazil; 3 Behavior and Psychobiology Graduate Program, Biosciences Center, Federal University of Rio Grande do Norte, Natal, Brazil; 4 Department of Botany and Zoology, Stellenbosch University c/o Sea Search Reserch and Conservation, Stellenbosch, South Africa; 5 Colegio de Ciencias Biológicas y Ambientales and Galápagos Science Center, Universidad San Francisco de Quito, Quito, Ecuador; 6 Acoustic Ecology Program, CETACEA, Proyecto, Ecuador; 7 Applied Ecology and Conservation Lab, State University of Santa Cruz, Ilhéus, Brazil; 8 Department of Biological Science, State University of Santa Cruz, Ilhéus, Brazil; 9 Laboratory of Acoustics and Environment, University of Sao Paulo, Sao Paulo, Brazil; 10 Acoustic Ecology and Animal Behaviour Laboratory, Universidade Federal do Recôncavo da Bahia, Cruz das Almas, Brazil; 11 Centre for Marine Science and Technology, Curtin University, Bentley, Australia; 12 Graduate Program in Ecology and Biodiversity Conservation, State University of Santa Cruz, Ilhéus, Brazil; 13 Parque Científico e Tecnológico do Sul da Bahia, Ilhéus, Brazil; 14 NGO Centro Ballena Azul, Universidad Austral de Chile, Valdivia, Chile; 15 Instituto de Ciencias Marinas y Limnológicas, Universidad Austral de Chile, Valdivia, Chile; 16 Center for Oceanographic Research COPAS Sur-Austral and COPAS Coastal, University of Concepción, Concepción, Chile; 17 Departamento de Oceanografía, Facultad de Ciencias Naturales y Oceanográficas, University of Concepción, Concepción, Chile; 18 Centro de Estudios Avanzados en Zonas Áridas (CEAZA), La Serena, Chile; 19 Helmholtz Institute for Functional Marine Biodiversity, Carl Von Ossietzky University Oldenburg, Oldenburg, Germany

**Keywords:** Conservation biology, Population dynamics, Behavioural ecology

## Abstract

The Atlantic sector of the Southern Ocean (ASSO) has one of the highest densities of Antarctic krill (*Euphausia superba*) compared to other polar and subpolar regions, which attracts migratory baleen whale species to aggregate in this area for feeding. Humpback whales (*Megaptera novaeangliae*) also sing extensively while on the Southern Ocean feeding grounds which allows for the exploration of song similarity between feeding grounds and breeding populations which helps to understand population mixing. The results of comparative song analyses between the ASSO and the Ecuadorian and Brazilian breeding populations and recordings from the Chilean, South African and Namibian migration routes/mid-latitude feeding grounds revealed that individuals from at least three humpback whale breeding populations most likely migrate to shared feeding grounds in the ASSO. Humpback whales from different populations potentially mix at different times (i.e., years) at feeding hotspots in variable locations. The ASSO seems to provide sufficient prey resources and seems to present an important area for both cultural and maybe even genetic exchange between populations supporting the maintenance of large gene pools. Assuming that multi-population feeding hotspots are also suitable habitat for krill and other krill-dependent predators, these areas in the ASSO should be carefully managed integrating population, ecosystem and fisheries management.

## Introduction

Humpback whale (*Megaptera novaeangliae*) populations of the Southern Hemisphere are known to aggregate in low latitude breeding areas which are confined by continental barriers (at least in one longitudinal direction)^1^. Due to maternally directed site fidelity, humpback whales usually return every year to their natal breeding ground, dividing Southern Hemisphere humpback whales into at least six distinct breeding populations^1^. The connectivity among these breeding populations usually correlates with the geographical distance between the areas, but most likely also depends on the degree of longitudinal movements of individuals within the Southern Ocean^2–5^. Humpback whales are flexible in their ecological requirements and are able to adapt to environmental change with alternative migration and feeding strategies^5–9^. Specific feeding areas, for example, can be visited during most years, but are abandoned during years with exceptional climatic conditions^10^. This migratory plasticity facilitates the mixing of populations within feeding aggregations allowing for cultural and genetic exchange^3,5,11,12^.

Baleen whale populations, and particularly their recovery from past whaling depletion, are managed by the International Whaling Commission (IWC). The IWC lists the identification of breeding/feeding ground migratory linkages and connections as a priority research topic to improve conservation and management efforts for Southern Hemisphere humpback whales^13^. These linkages can be identified by satellite tagging individual whales, using photographic mark-recapture, or comparing genetic markers or songs of individual whales between regions^5,14,15^. The songs of male humpback whales can be recorded mainly during late autumn, winter, and early spring (i.e., shortly before, during and shortly after the breeding period of humpback whales) and are highly similar among males within a breeding population, but are (to some extent) distinct among males from different breeding populations^14,16^. Especially for areas with restricted access, such as the Southern Ocean, the possibility of investigating migratory linkages between breeding and feeding grounds through the comparison of population-specific humpback whale songs is a great advance for humpback whale population monitoring^4,12,17,18^. The availability of long-term passive acoustic monitoring data from the Southern Ocean and the discovery that humpback whales sing extensively while on the Southern Ocean feeding grounds and migration routes^12,19–21^ now allows for the exploration of song similarity between breeding and feeding grounds which can be used as an indicator for population mixing^4,17,22^.

The Atlantic sector of the Southern Ocean (ASSO) is regularly frequented by humpback whales and its Northern limit (i.e., in the vicinity of the Antarctic Polar Front) is especially suitable feeding habitat for humpback whales^10,23–25^. At least two distinct humpback whale breeding populations have been found to visit and overlap within the ASSO^5,12,26^ and a high similarity in songs from South Atlantic breeding areas suggests occasional mixing of populations, potentially on a common feeding ground^4^. To gain a more comprehensive understanding of how many and which humpback whale populations mix in the ASSO, this study aims to conduct comparative analysis of humpback whale songs from the ASSO and available temporally matching song recordings from the Ecuadorian (breeding stock G^27^) and Brazilian (breeding stock A^27^) breeding grounds and the Chilean, Namibian, and western South African migration routes/mid-latitude feeding grounds (of breeding stocks G and B, respectively; assumed migratory connections by the IWC^27^). We discuss the temporal flexibility in the migration and mixing of humpback whales from three populations and highlight the importance of mixing areas for the viability of recovering baleen whale populations and Southern Ocean pelagic ecosystem.

## Material and methods

### Data and processing

**Figure 1:**
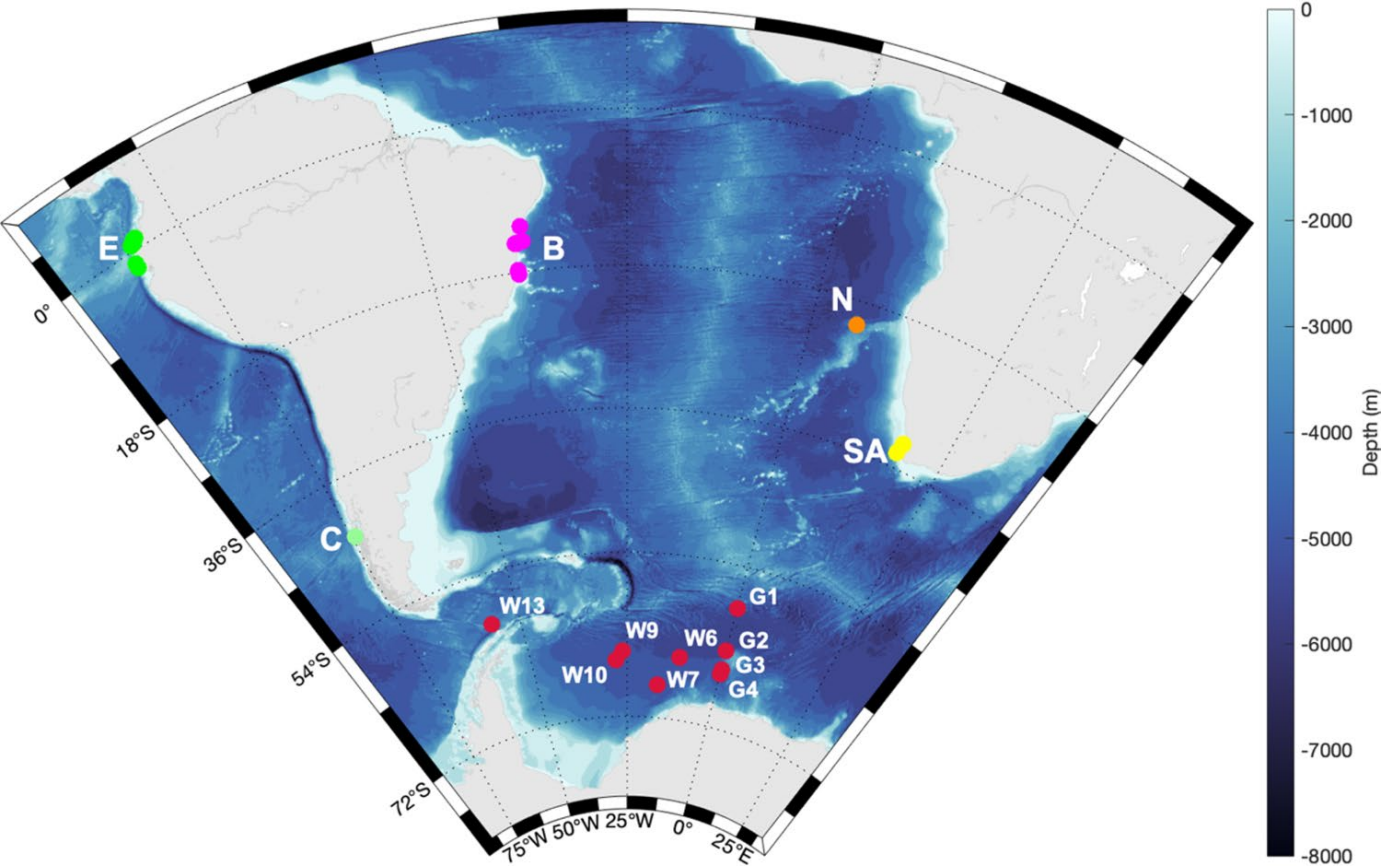
Bathymetric map with recording locations in the Atlantic, Pacific and Southern Ocean: Greenwich Meridian (G1–4), Weddell Sea (W6–13), Ecuador (E, green), Chile (C, light green), Brazil (B, pink), Namibia (N, orange), South Africa (SA, yellow). Recording locations marked with a red dot, in sum, represent the recordings from the Atlantic sector of the Southern Ocean (ASSO). Map was generated with M_MAP in MATLAB^34^.

A prerequisite for comparative song analysis is the temporal proximity of the song recordings which are to be compared (i.e., songs from the same years or from previous or following seasons) due to the evolutionary nature of humpback whale songs^28,29^. Therefore, we gathered feeding ground, breeding ground, and migration route/mid-latitude feeding ground song recordings from 2011 to 2019 for the comparative analyses. Recordings were conducted either with stationary autonomous recording devices which were moored to the seafloor or with portable hydrophones which were submerged from a boat in the vicinity of humpback whales^12,30–32^. We extracted high quality humpback whale songs (i.e., signal-to-noise ratio ≥ 10 dB and at least two discernible distinct themes, meaning two distinct combinations of vocalizations which are usually repeated multiple times and therefore form a rhythmic song^12,33^) from passive acoustic recordings collected in the ASSO, off Ecuador, Chile, Brazil, Namibia, and South Africa using a range of recording setups (Fig. 1, Table 1). In the two breeding areas, Ecuador and Brazil, recordings were conducted at multiple locations which sometimes changed during recordings (due to the boat drifting with the current), therefore the larger area where recordings were conducted is defined by the latitude and longitude limits provided in Table 1.

**Table 1:** Details of passive acoustic recordings from the six regions: Atlantic sector of the Southern Ocean (ASSO), Ecuador, Chile, Brazil, South Africa, Namibia.

Location	Latitude	Longitude	Recording device	Digitalization
ASSO	66° 36.45′ S 66° 30.708′ S 66° 2.01′ S 65° 58.092′ S 63° 59.846′ S 61° 1.071′ S 59° 2.823′ S	27° 7.26′ W 0° 1.512′ E 0° 3.117′ E 12° 15.12′ W 0° 1.842′ E 55° 58.686′ W 0° 5.775′ E	SonoVaults (Develogic GmbH, Hamburg)	5333–9600 Hz
Ecuador	1° 7.3293′ N to 1° 48.5′ S	79° 43.9392′ W to 81° 6.92′ W	H2a-XLR omnidirectional hydrophone DolphinEar/PRO hydrophone	44,100 Hz
Chile	43° 31.889′ S	74° 26.488′ W	Marine Autonomous Recording Units (MARUs)	2000 Hz
Brazil	14° 28.6671′ S to 18° 13.9536′ S	38° 0.5052′ W to 39° 0.6865′ W	Archival bottom-mounted Oceanpods Tascam DR40/HTI 96 min Zoom H4nPro/Aquarium Audio H2a M-Audio Inc. Micro track II	11,000–48,000 Hz
South Africa	34° 9.3396′ S 32° 46.1346′ S	18° 17.7′ E 17° 52.0656′ E	SoundTrap (Ocean Instruments, NZ)	48,000 Hz
Namibia	20° 57.90′ S	5° 58.82′ E	SonoVaults (Develogic GmbH, Hamburg)	5333 Hz

Latitudes and longitudes specified with “to” describe an area where recordings have been conducted at many different locations.

Humpback whale vocalizations were manually logged within the spectrograms in Raven Pro (Hann Window, 1025–8057 window size, 80% overlap, 2048–8192 DFT size^35^). Logged vocalizations were manually classified into distinct unit types and subtypes (call types: CT followed by a number; subtypes: CT followed by a number and a lower case letter) according to the following criteria: (1) differentiation of tonal or broadband characteristics, (2) duration, (3) time–frequency slope and (4) frequency range (vocalizations having the same characteristics regarding criteria (1) to (3) but were encountered in different frequency ranges, were classified into subtypes). Within a humpback whale song sequence, phrases were logged and classified according to unit repetition following the recommendations of Cholewiak et al.^33^. Phrase types were identified with an uppercase letter (indicating the 1st unit type), a lowercase letter (indicating the combination of following unit types) and a sequence of numbers (indicating the number of repetitions of each unit).

The manual subjective analysis of unit repertoire was tested in terms of robustness by applying an automated classification approach to a subset of units (436 exemplar units with at least 20 exemplars per unit type sampled from different locations and days of recording^36^). We computed 44 different acoustic metrics for every extracted unit (i.e., 3 s sound file decimated to 5000 Hz to ensure comparability). The 44 metrics can be described as belonging to either of these three categories: (1) indices based on different algorithms to compute acoustic complexity, entropy or diversity (acoustic indices), (2) metrics measuring amplitude or background patterns (energy metrics), and (3) metrics computing ratios between acoustic activity over time and frequency bands (ratio metrics). Details on the acoustic metrices used and the process of computation for the 436 sound examples can be found in Schall et al.^36^. The 44 acoustic metrices for each extracted unit were used in a random forest supervised machine learning approach^36^ to discriminate between manually classified unit types.

To assess inter- and intra-individual song differences, first, individual singers were differentiated. For recordings made with a dipping hydrophone, individual singers were differentiated in the field by human observers. For autonomous recordings, spatio-temporal assumptions were applied to allocate presumed individual singers (for details on assumptions, see^12^). Second, the start and end of an explicit song had to be defined. Inspecting our song sequence data for common patterns, the most sensible definition for song was the complete rendition of all unique theme types per song sequence to form an explicit humpback whale song^33^.

### Song repertoire and structure comparison

The phrase repertoires of geographic groups (i.e., ASSO, Ecuador, Chile, Brazil, Namibia, South Africa) were first clustered by year, and in case of the ASSO, by song group with the data from 2013 split into song group 1 and 2 because these song groups had 0% similarity in previous analyses^12^. Repertoires of these clusters were compared by applying the Dice Coincidence Index (DCI) with a custom-written script in R^37,38^:

$$DCI=\frac{2A}{B+C},$$

with *A* being the number of shared phrase types between a pair of singers, *B* and *C* being the number of phrase types of each singer, respectively. The resulting similarity matrix was subjected to a hierarchical cluster analysis in R^38^ using the “average linkage” method and the output was visualized in a dendrogram. Hierarchical clustering was bootstrapped (1000 times) with the R function ‘pvclust’^39^ to generate approximate unbiased (AU) values with AU values exceeding 95% indicating dendrogram divisions that are likely to occur.

To compare the song structure (the order of themes within a song) among geographic groups, the sequences of phrases were transcribed to sequences of themes (ignoring the repetition of phrases) and a set median string was chosen for each individual singer. The set median string was defined as the sequence of themes which had the highest similarity to all sequences of themes of a given set. The similarity between sequences was calculated by applying the Levenshtein Distance Similarity Index (LSI) in MATLAB^40,41^:

$$LSI\left(a,b\right)=1-\frac{\mathrm{min}\left(I+D+S\right)}{\mathrm{max}\left[L\left(a\right),L\left(b\right)\right]},$$

with *a* and *b* being the two theme sequences, *I* being insertions, *D* being deletions, *S* being substitutions and *L* being the length of the respective sequence. In the following, the set median strings of the breeding grounds (i.e., Ecuador and Brazil) were clustered per year and a set median string was chosen from each location-year cluster applying the same method as above. The songs recorded in the ASSO and on the migration routes/mid-latitude feeding grounds were all included in the comparative analyses (without reduction to a representative theme sequence per year) due to the unknown number of breeding populations that potentially contributed to the recordings.

Humpback whale songs from the distinct geographic groups were compared for each year of ASSO song recordings including also breeding ground or migration route/mid-latitude feeding ground songs from the year before or after, by applying the LSI to pairs of individuals/clusters with the R function ‘stringdist’^42^. The resulting similarity matrix was subjected to a hierarchical cluster analysis using the “average linkage” method, the output was visualized in a dendrogram, and hierarchical clustering was bootstrapped (1000 times)^38,39^.

## Results

**Figure 2:**
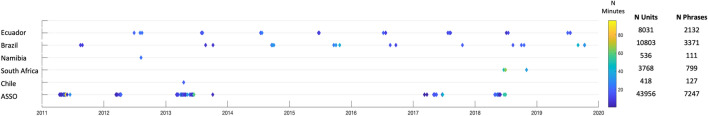
Timeline of song recordings from the six recording locations. Each diamond marks the date of recording of one or multiple individual singers. The color of the diamond represents the number of minutes of analyzed song. For each location, the number of classified units and phrases is listed next to the timeline.

We analyzed humpback whale songs from the ASSO, Brazil, Ecuador, South Africa, Chile, and Namibia for on average 3 individual singers per year and location (between one and 25 individual singers per year and location; see supplementary material I for list of analyzed songs). Fewer individual singers per year and location were only considered if more data was not available (Fig. 2). In the case of the ASSO, songs from as many individual singers as possible were analyzed (10–25 per year) because an unknown number of breeding populations could have contributed to these songs. In the ASSO, data collection in 2014 failed so that no acoustic recordings could be analyzed and in the years 2015 and 2016, passive acoustic data were collected, but did not contain any humpback whale songs (see^12^ for more details and possible explanations). From the different individual singers between one and 95 min (on average 16.4 min) of humpback whale song were analyzed, depending on the availability of good quality song sequences (Fig. 2). In total, 67,512 song units were classified into 15 distinct unit types, plus seven subtypes, and 13,787 phrases were classified into 114 distinct phrase types (see supplementary material II for catalogue of unit and phrase types). The level of agreement between the manual unit classification and the result of the supervised machine learning approach was high with a general ‘Out-of-bag’ misclassification rate of 16% indicating a robust differentiation of units, phrases, themes and songs (see^36^ for more detailed results).

**Figure 3:**
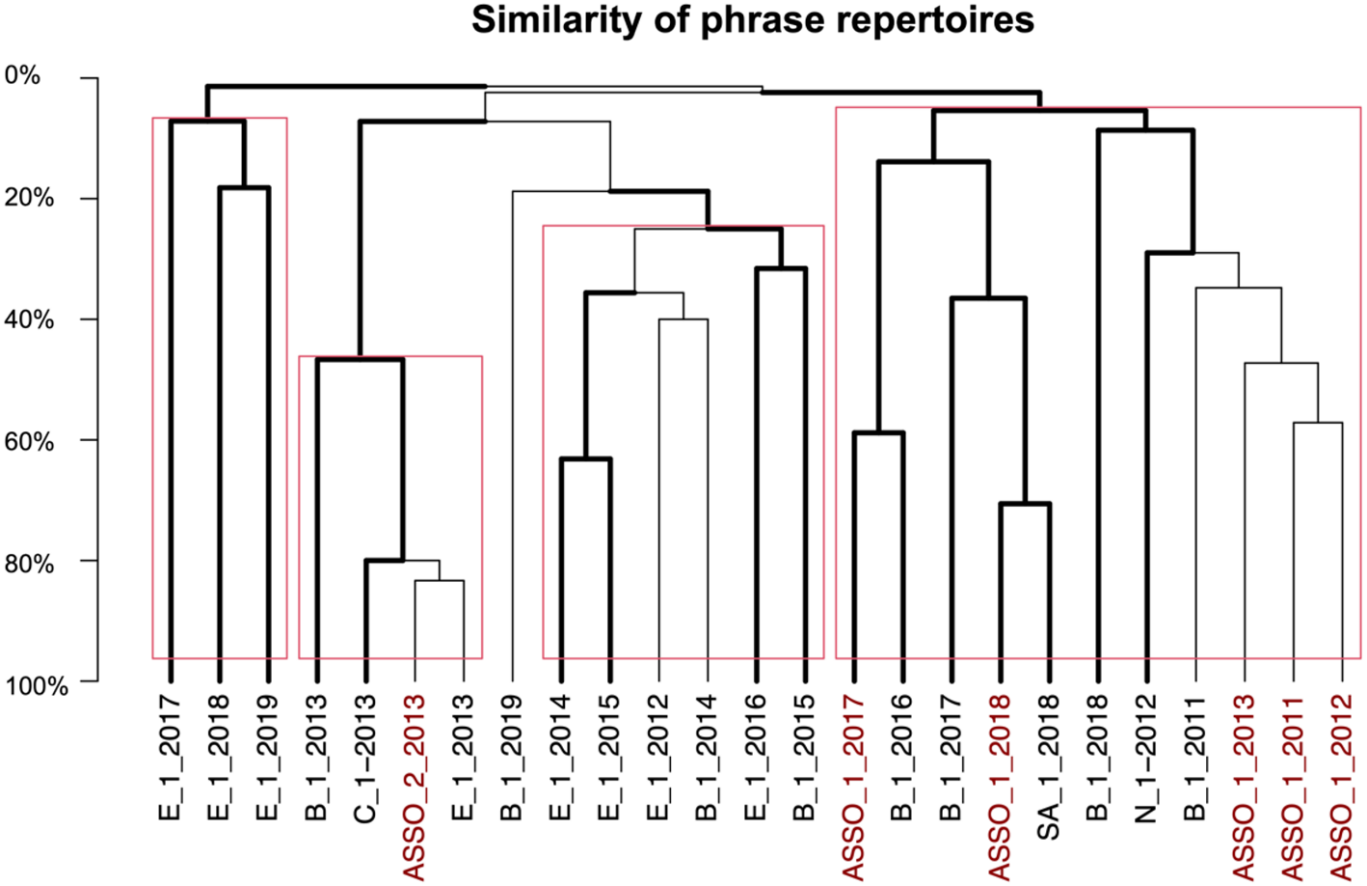
Bootstrapped dendrogram from hierarchical clustering of the similarity of phrase repertoires (dice coincidence index) for humpback whale songs from the Atlantic sector of the Souther Ocean (ASSO), the Ecuadorian breeding population (E), the Brazilian breeding population (B), and the South African (SA), Chilean (C) and Namibian (N) migration routes/mid-latitude feeding grounds for all recording years. Phrase repertoires were compared as summarized repertoires per location and year. ASSO recordings from 2013 are represented as two repertoires, due to the clear differentiation of two song groups^12^. The names on each branch indicate the location (e.g., ‘E’ for Ecuador, ‘B’ for Brazil, and ‘SA’ for South Africa), the repertoire ID (e.g., ‘1’, ‘2’), and the year (e.g., ‘2017’) to identify the respective phrase repertoire. Bold lines indicate divisions that were likely to occur (i.e., approximate unbiased value > 95%) and red rectangles indicate clusters of significant probability.

Humpback whale songs from the ASSO shared phrase types with all the breeding and migration route/mid-latitude feeding ground songs included in the comparative analyses (Ecuador, Chile, Brazil, South Africa, and Namibia). The comparison of phrase repertoires revealed a high overlap (> 80%) between the ASSO song group 2 repertoire recorded off Elephant Island and the Ecuadorian and Chilean repertoires in 2013 (‘ASSO_2_2013’, ‘E_1_2013’, and ‘C_1_2013’; Fig. 3). Humpback whale songs from the ASSO in 2017 shared almost 60% of the phrase types with the songs recorded off Brazil in 2016 (‘ASSO_1_2017’ and ‘B_1_2016’; Fig. 3), while the ASSO songs from 2018 shared 70% of the phrase types with the South African songs from the same year (‘ASSO_1_2018’ and ‘SA_1_2018’). The ASSO repertoires from 2011, 2012, and 2013 had some overlap (> 27%) with the repertoires recorded off Namibia in 2012 and Brazil in 2011 (‘ASSO_1_2011’, ‘ASSO_1_2012’, ‘ASSO_1_2013’, ‘B_1_2011’, and ‘N_1_2012’; Fig. 3). Brazilian repertoires from 2014, 2015, 2019 and repertoires from Ecuador 2012, 2014, 2015, 2016, 2017, 2018, 2019 clustered separately indicating no or little (< 10%) overlap with phrase repertoires from the ASSO.

**Figure 4:**
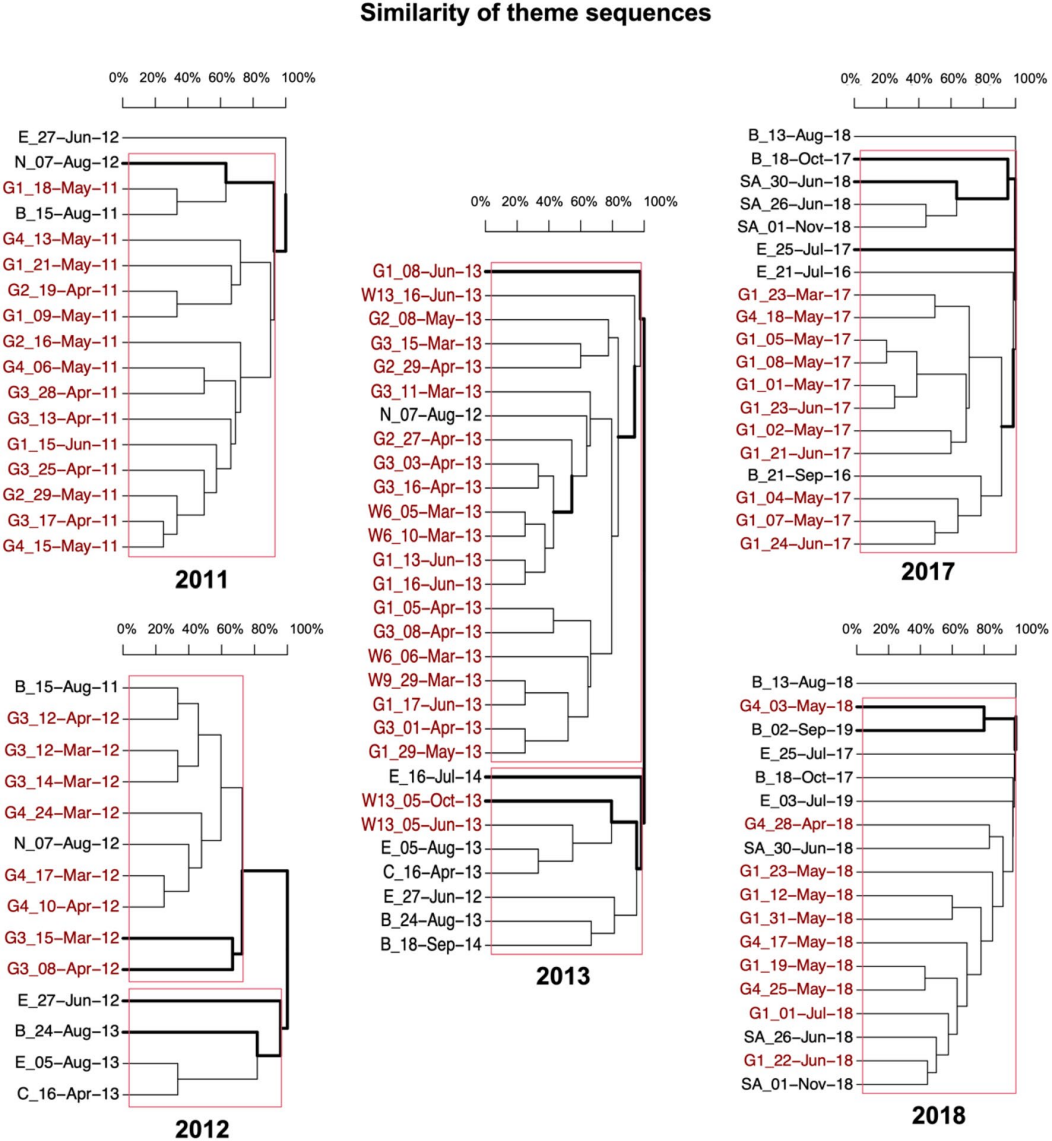
Bootstrapped dendrograms from hierachical clustering of similarity of theme sequences of humpback whales songs (Levensthein distance similarity index) from the Atlantic sector of the Souther Ocean (ASSO: reprsented by recording positions G and W), the Ecuadorian breeding population (E), the Brazilian breeding population (B), and the South African (SA), Chilean (C) and Namibian (N) migration routes/mid-latitude feeding grounds for the five different years of song recordings from the ASSO. Names on each branch belong to individual singers in case of the ASSO and migration route/mid-latitude feeding ground recordings, or representative theme sequence in case of breeding population recordings encoded with the name of the recording position (first 2–3 symbols, e.g., ‘W13’, ‘G4’, ‘SA’,…) and the date of the recording (last 9 symbols, e.g., ‘28-Apr-18’, ‘01-Nov-18’,…). Bold lines indicate divisions that were likely to occur (approximate unbiased value > 95%) and red rectangles indicate clusters of significant probability.

**Figure 5:**
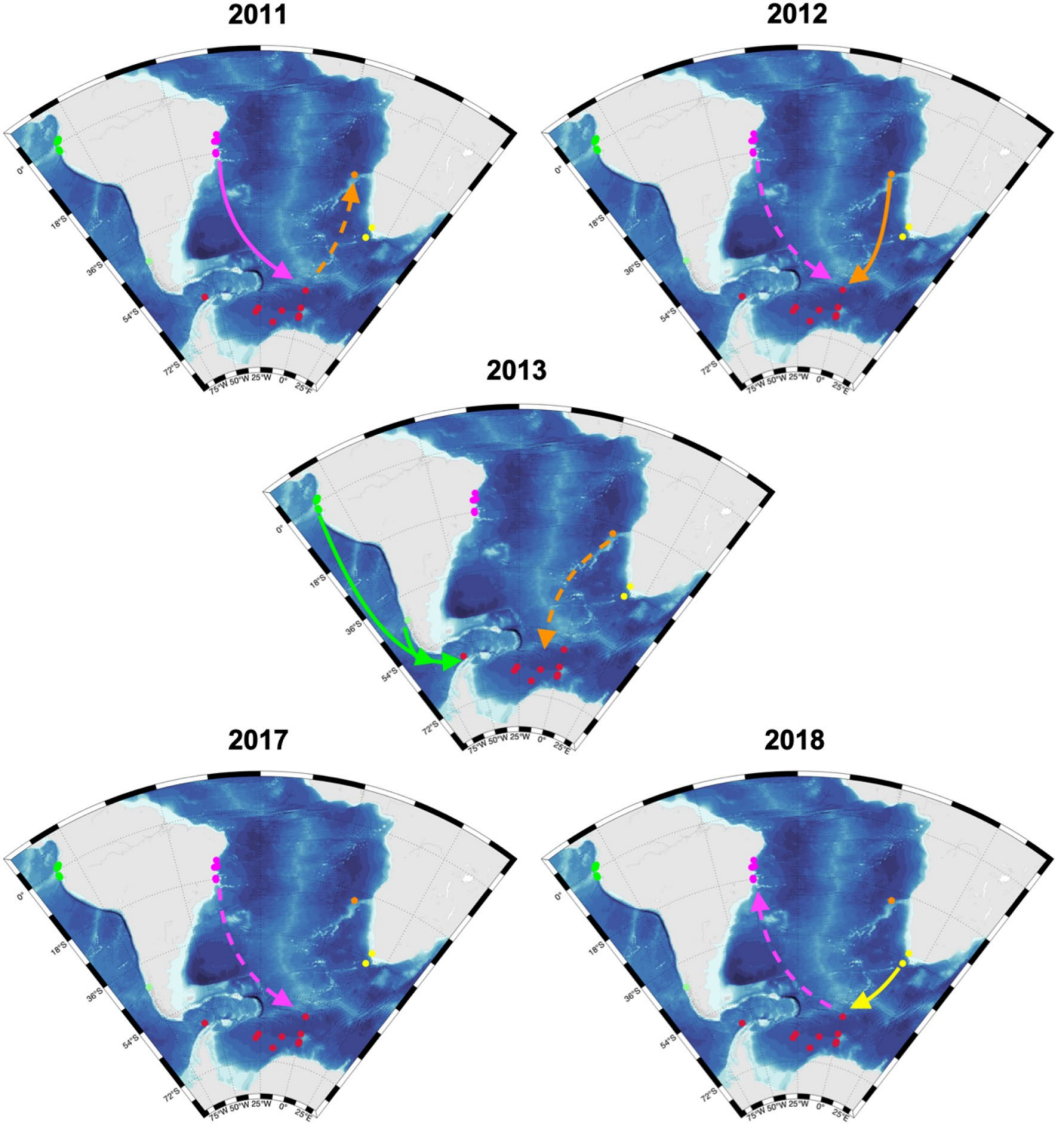
Schematic illustration of potential humpback whale song transmission pathways between the Atlantic sector of the Souther Ocean (ASSO: red dots) and the Ecuadorian breeding population (green dots), the Brazilian breeding population (magenta dots), and the Chilean (light green dots), South African (yellow dots), and Namibian (orange dots) migration routes/mid-latitude feeding grounds indicated by comparative song analyses. Red dots indicate recording positions in the ASSO. Arrows illustrate the likely pathways of acoustic contact between humpback whales recorded at the different locations for the same year. Southward facing dashed arrows illustrate likely acoustic contact either during the same year or the year before and northward facing dashed arrows illustrate a likely acoustic contact either in the same year or the year after. Maps were generated with M_MAP in MATLAB^34^.

Song structures, represented by theme sequences in the ASSO also showed similarities to songs recorded at all locations included in the comparative analyses, depending on the year of recording. In 2011, the songs recorded from one individual in the ASSO were 70% similar to the songs recorded off Brazil in the same year and 40% similar to the songs recorded off Namibia the year after (Fig. 4). This degree of similarity indicates that humpback whales from the Brazilian breeding population migrated to the ASSO feeding area, more specifically to the area around the Greenwich Meridian, and returned to the waters off Brazil in winter (Fig. 5). This is also evident from the comparative analyses of the 2012 data, where the 70% of song similarity between the Greenwich recordings and the Brazilian recordings from 2011 indicate acoustic contact in 2012 or 2011 (Figs. 4, 5). Humpback whales from the offshore Namibian migration route, visited the ASSO in 2012 and potentially also in 2011 and/or 2013, indicated by theme sequence similarities between 30 and 50% (Figs. 4, 5). The hierarchical cluster of the 2012 data also shows a connection between Ecuadorian and Brazilian song with a higher similarity between the Brazilian and Ecuadorian song from 2013 than between the Ecuadorian song from 2012 and 2013 (Fig. 4), which implies an event of acoustic contact between these populations in 2012, potentially in the ASSO. The 2013 data shows that the songs of two individuals recorded off Elephant Island (i.e., ‘W13_05-Jun-2013’ and ‘W13_05-Oct-2013’ representing song group 2 from Schall et al.^12^) were the most similar to the songs recorded off Ecuador and Chile in the same year. This indicates that humpback whales from the Ecuadorian breeding population and Chilean migration route/mid-latitude feeding ground visited the area around Elephant Island, at least during this particular year (Figs. 4, 5). All other song recordings from the ASSO in 2013 were clustered together with the song recording from Namibia in 2012 and separately from Ecuadorian, Chilean, and Brazilian song recordings (Fig. 4). Therefore, only humpback whales from the west African breeding population (here represented by the offshore Namibian migration route recordings) contributed to the prevalent song group 1 recorded throughout the entire ASSO (i.e., song group 1 from Schall et al.^12^). All the songs recorded in the ASSO during 2017 and 2018 were partly similar (i.e., 20%) to Brazilian songs from 2016 and 2019, but almost completely different (i.e., < 5%) from the Brazilian songs from 2017 and 2018 (Fig. 4), indicating acoustic contact between the Brazilian breeding population and the ASSO before 2017 and after 2018 (Fig. 5). The songs recorded in the ASSO in 2018 instead were similar (17–57%) to the songs recorded off South Africa during the same year, indicating a direct migratory link between these two locations (Figs. 4, 5).

## Discussion

Overall, the comparative song analyses showed that humpback whales from the west African (represented by the animals recorded off South Africa and Namibia), Ecuadorian and Brazilian breeding populations had acoustic contact to humpback whales recorded in the ASSO, most likely because at least some members of these breeding populations migrate to a shared feeding ground in the ASSO. In the case of the ASSO, the comparative analysis of songs seems to be a promising and useful tool to study migratory connections and mixing patterns among breeding populations.

These results confirm the previous suggestion by Darling and Sousa-Lima^4^ and the findings presented in Schall et al.^12^ of the presence of humpback whales from at least two breeding populations feeding in the ASSO, and presents evidence for the migration of at least three breeding populations to the ASSO feeding area. Indications of mixing in the ASSO were observed in multiple years and therefore was not a single event (see also^4,5^). For humpback whales from the South Atlantic breeding populations, the ASSO has been assumed to be the primary feeding ground^43–45^. Additionally, the results confirm the suggested cultural exchange among humpback whales from the Ecuadorian breeding population and humpback whales from various South Atlantic breeding populations which all migrate to the ASSO where their habitat ranges overlap spatially^5^. Within this context, it is also possible that humpback whales from other breeding populations than those included in these comparative song analyses (e.g., humpback whales breeding in the Southwestern Indian Ocean), migrate to the ASSO and contributed to the encountered variabilities in song recordings. Future studies could examine circum-Antarctic patterns of song exchange among humpback whale breeding populations at a broader scale by applying the same methods which are presented here. Cultural exchange in terms of song learning requires whales to be within a relatively short distance of each other. Maximum detection range of songs is estimated to be 50 km in the ASSO^25^ although animals likely need to be < 10 km apart to be able to fully perceive all frequency components of the song^46^. These values suggest that individual whales from different populations must be within the required spatial vicinity of each other for song exchange, a behavior that also ultimately favors crossbreeding among populations^11^.

Mixing patterns seem to be temporally variable, meaning that whales from different populations potentially mix at different times (i.e., years or months) at feeding hotspots of variable locations. Based on the acoustic similarities, humpback whales from the Brazilian breeding population, for example, are likely to have visited the area around the Greenwich Meridian in the ASSO during 2011, 2016, and 2019. However, no similarity between songs from Brazil and the ASSO was found for 2013 and 2018. The flexibility of these mixing processes is most likely connected to variations in migratory patterns driven by spatio-temporal changes in prey availability^5,47,48^. Optimizing their energy budgets, humpback whales from different breeding populations are likely to migrate to those areas with sufficient prey availability that lie closest to the respective breeding ground^49^. Baleen whales are thought to employ a multi-modal sensory system combining magnetoreception, somatosensory perception of oceanographic conditions, chemosensory cues as well as acoustic perception of conspecifics or other marine animals to find prey hotspots^50,51^. Humpback whales can detect and localize social vocalizations and songs of conspecifics over tens of kilometers^52,53^ which allows humpback whales to relatively flexibly navigate to ephemeral prey hotspots following acoustic way markers. Humpback whale song produced at feeding hotspots might serve the purpose of attracting more individuals to these hotspots in order to promote nutrition of females and calves (i.e., to promote receptivity in females and assure survival of kin) and increase chances of reproduction with potentially receptive females^16^. Additionally, individual whales were also observed to migrate to or towards a different breeding ground, where cultural and genetic exchange could take place^54^; www.happywhale.com^55–57^.

Within the Southern Ocean, a high krill availability in the ASSO may attract humpback whales from different breeding populations and favors mixing among the different populations^11^. On average, the polar and subpolar regions of the South Atlantic Ocean (i.e., including the ASSO) have the highest densities of Antarctic krill (*Euphausia superba*) on a circumpolar scale^58,59^. The locations of krill hotspots in the Southern Ocean vary on intra- and interannual temporal scales and are driven by sea ice, oceanographic, and climatological dynamics^60–62^. In contrast to the whales of the Northern Hemisphere, migratory baleen whales from the Southern Hemisphere are not restricted by continental barriers at high latitudes and can therefore choose feeding locations over a large longitudinal range^11,15,63^. As our results suggest, even humpback whales from two different ocean basins migrate to the ASSO to feed and potentially reproduce. This mixing of multiple humpback whale populations in the ASSO is also supported by genetic analyses of individuals sampled in the ASSO in comparison to individuals sampled in the rest of the Southern Ocean, where no significant genetic differentiation (i.e., mitochondrial and microsatellite) was found between areas^11^.

Humpback whales from most Southern Hemisphere breeding populations are recovering well from past overexploitation through industrial whaling^27^ and areas, such as the ASSO where multiple breeding populations feed in mixed aggregations could be of key importance to the positive population trends recorded during the past decade. Two important factors which ensure the prosperity of a population are linked to the ASSO: (1) the ASSO provides sufficient prey resources to allow population growth^64–67^, and (2) the ASSO is an important area for both cultural and maybe even genetic exchange between populations supporting the maintenance of large gene pools which increase the populations’ resilience to environmental change^11^. Our results clearly suggest that multiple humpback whale populations visit the ASSO during the feeding season and that cultural exchange in form of song learning is taking place in this region.

Combining these multiple lines of evidence, the ecological relevance of the ASSO for humpback whales from multiple populations is clear, while it seems also clear that other locations can become relevant in this context when considering potential future environmental changes, e.g., more frequent El Niño events^68^. Contemporary multi-population humpback whale hotspots in the ASSO, as the eastern and western edges of the ASSO along the polar front (i.e., areas around the recording locations G1 and W13 from this study), should be carefully managed by integrating population, ecosystem and fishery management strategies led by the IWC and the Commission for the Conservation of Antarctic Marine Living Resources (CCAMLR). Since the spatiotemporal distribution of humpback whales in the ASSO is most likely driven mainly by the availability and distribution of their primary prey species^69^, it is safe to assume that humpback whale hotspots reflect areas with high krill densities (although whales may target specific age and size classes representing only part of the krill population^70^). In addition to the importance of the ASSO for multiple humpback whale populations, humpback whale feeding hotspots in the ASSO could therefore also indicate areas of importance for Antarctic krill, the keystone species of the Southern Ocean, and other krill-dependent predator species^71^. Due to this overlap and the role of baleen whales in structuring the Southern Ocean pelagic ecosystem^72^, the creation of a marine protected area (i.e., the Weddell Sea MPA^73^) including humpback whale hotspots could be an effective management strategy beneficial to single species, ecosystem processes, as well as fisheries^74^.

## Supplementary Information

Supplementary Information 1.

Supplementary Information 2.

## Data Availability

Analyses reported in this article can be reproduced using the data provided by Schall (2021) at Data Dryad: https://datadryad.org/stash/share/Vfg14Wkti-rXiDZWqIAJX7cdHGxFqUiPUUOWIWx5h6E.
