# Supplementary material for: Song recordings suggest feeding ground sharing in Southern Hemisphere humpback whales
Source: Sci Rep. 2022 Aug 17;12:13924. doi: 10.1038/s41598-022-17999-y (PMC9385655; doi:10.1038/s41598-022-17999-y)
Supplement: Supplementary file 1 — Supplementary Information 1. [file 41598_2022_17999_MOESM1_ESM.pdf]

Song recordings suggest feeding ground sharing in Southern Hemisphere humpback whales

Authors
Elena Schall, Divna Djokic, Erin C. Ross-Marsh, Javier Oña, Judith Denkinger, Julio Ernesto Baumgarten, Linilson Rodrigues Padovese, Marcos R. Rossi-Santos, Maria Isabel Carvalho Goncalves, Renata Sousa-Lima, Rodrigo Hucke-Gaete, Simon Elwen, Susannah Buchan, Tess Gridley, Ilse Van Opzeeland

-Supplementary Material 1-

SingerID	Median String
**E_27-Jun-12**	Dj Ja Ia Ic Cg As Ar Au
**E_02-Aug-12**	Dj Ja Ia Ic Cg Ar As Au
**E_13-Aug-12**	Dj Ja Ic Ia Cg Ar As Au
**E_30-Jul-13**	Ec Ed Gb Ga Dj
**E_05-Aug-13**	Ec Ed Gb Ga Dj Ha
**E_06-Aug-13**	Ec Ed Gb Ga Dj Ha
**E_14-Jul-14**	Ar Kc Av Fb Ja Jb Id
**E_16-Jul-14**	Ar Kc Av Fb Jb Ja Id Ia
**E_23-Jul-14**	Ar Av Kc Fb Jb Ja Ia Id
**E_22-Jun-15**	Ar Ja La Aw Ax Kd Fc Jb Id Ia
**E_25-Jun-15**	Ar Aw La Ax Kd Fc Jb Id Ia
**E_26-Jun-15**	Ar Aw La Ax Kd Fb Jb Id
**E_08-Jul-16**	Ar Gl Ay Na Lb Ma Ia Kd
**E_21-Jul-16**	Ar Lb Ma Ia
**E_23-Jul-16**	Ar Gl Ay Na Lb Ma Ic Ia
**E_25-Jul-17**	Ma Ib Ie Ih Ig Ii
**E_02-Aug-17**	Ma Ib Ie Ih If Ig Ii
**E_10-Aug-17**	Ma Ib Ie If Ih Ig Ii
**E_05-Jul-18**	Ij Ik
**E_17-Jul-18**	Dm Il
**E_03-Jul-19**	Ic Dm Dg Lc Ld Dl Ib
**E_17-Jul-19**	Ic Dm Dg Lc Ib
**E_18-Jul-19**	Ic Dm Dg Lc Ld Dl Ib
**E_19-Jul-19**	Ic Ib Dm Dg Lc Ld Dl
**B_21-Sep-16**	Bq Df Ef Ee Ia Be
**B_21-Sep-16**	Df Ef Ee Ia Be Bq
**B_18-Oct-17**	Dn Bh Bn Bm Bp Do Ia Bi Mb

**B_18-Oct-17**	Bn Bm Gc Bb Bo Bp Bh
**B_18-Oct-17**	Bn Bk Gc Bc
**B_18-Sep-14**	Ja Ec Eh Ar As Jb
**B_22-Sep-14**	Ar As Jb Ja Ec Eh
**B_20-Sep-15**	Bl Ar Ka Gj Kb Ia Ec Eh
**B_03-Oct-15**	Bl Be Ar Ka Gj Kb Ia Ec Eh
**B_25-Oct-15**	Bl Ka Gj Kb Ia Ec Eh
**B_13-Aug-18**	Db At Dj
**B_01-Oct-18**	Db Dj At
**B_02-Sep-19**	Df Ar Dk Gk
**B_15-Aug-11**	Aa Af Da Db Ba Bh
**B_27-Aug-11**	Ba Bh Aa Af
**B_24-Aug-13**	Ec Gb As Jb Ed
**B_07-Oct-13**	Gb Gm As Jb Ec
**B_18-Aug-16**	Ee Ia Be Bf Df
**N_07-Aug-12**	Aa Ak Bc Ba Ai
**SA_21-Jun-18**	Bb Gg Bh Bi Bc Bj Gh
**SA_30-Jun-18**	Bh Bc Bj Bb Gd
**SA_01-Nov-18**	Gd Ge Gg Bh Bi Bc Bj Bb Bk
**C_16-Apr-13**	Gb Ga Ha Ec
**G3_28-Apr-11**	Cb Cc Ca Ba Ea
**G2_16-May-11**	Cb Cc Ba Aa
**G3_13-Apr-11**	Cb Cc
**G3_17-Apr-11**	Ea Cb Ba Aa
**G2_19-Apr-11**	Ba Aa
**G3_25-Apr-11**	Ea Cb Ca Cc Ba
**G4_27-Apr-11**	Cb Cc
**G4_06-May-11**	Ea Cb Cc Ca
**G2_09-May-11**	Cb Cc
**G1_09-May-11**	Ba Ac Aa
**G2_12-May-11**	Cb Cc
**G4_13-May-11**	Ba Ac
**G4_15-May-11**	Ea Cb Cc Aa
**G3_17-May-11**	Aa Ea Cb Cc Ca Ba
**G1_18-May-11**	Ba Aa Ac Bb Ab
**G1_21-May-11**	Ba Aa Ac
**G2_29-May-11**	Cb Cc
**G1_15-Jun-11**	Cc Cb Ba Aa Ea
**G3_12-Mar-12**	Aa Ba
**G3_14-Mar-12**	Ac Aa Af Da De Ba
**G3_15-Mar-12**	Ac Aa Ba
**G4_17-Mar-12**	Aa Ai Ba

**G4_24-Mar-12**	Aa Ac Ba	
**G3_04-Apr-12**	Aa Ac Ba	
**G4_07-Apr-12**	Aa Ac Ba	
**G3_08-Apr-12**	Ba Aa	
**G4_10-Apr-12**	Aa Ai Aj Ba	
**G3_12-Apr-12**	Ba Aa Af Da	
**W6_05-Mar-13**	Aa Ai Ac	Song group 1
**W6_06-Mar-13**	Ad Aa Ac	
**W6_10-Mar-13**	Aa Ai Ak Ac	
**G3_15-Mar13**	Ac An Ak	
**W9_29-Mar-13**	Ap Aa	
**G3_31-Mar-13**	Aa Ai Ak Ac	
**G3_11-Mar-13**	Ai Ac	
**G3_01-Apr-13**	Ac Aa Ai	
**G3_03-Apr-13**	Aa Ai Aj Ac Ad	
**G1_05-Apr-13**	Ac Aq Aa Am Ai An Ak	
**G3_08-Apr-13**	Aq Aa Am Ai Ac	
**G3_13-Apr-13**	Aa Ai	
**G3_16-Apr-13**	Aa Ai An Ak Ac Ad	
**G2_20-Apr-13**	Aa Ai Ac	
**G2_27-Apr-13**	Aa Ap Ai Aq Ac	
**G2_29-Apr-13**	Aa Ac Ai Ak Ap	
**G2_08-May-13**	Ap Aa	
**G1_21-May-13**	Aa Ai Ac	
**G1_29-May-13**	Aa Ai Ac	
**G1_30-May-13**	Aa Ai Ac	
**W13_05-Jun-13**	Ga Ha Ec Ed Gb	Song group 2
**G1_08-Jun-13**	Cb Fa Ba Ca Aj Ak	Song group 1
**G1_13-Jun-13**	Aa Ac	
**G1_16-Jun-13**	Aa Ai Ac Aq	
**W13_16-Jun-13**	Ai Ap	
**G1_17-Jun-13**	Ap Aa Ai	
**W13_05-Oct-13**	Ed Gb Ga	Song group 2
**G4_09-Mar-17**	Df Ee	
**G1_23-Mar-17**	Bf Bd Bg Ee	
**G1_01-May-17**	Bd Be Df Ee	
**G1_02-May-17**	Bd Df Ee	
**G1_04-May-17**	Be Df Ee Bf	
**G1_05-May-17**	Bd Bg Df Ef Ee	
**G1 07/05/17**	Df Ee	
**G1_08-May-17**	Bg Df Ee	
**G4_18-May-17**	Df Bd Be Ee	

**G1_21-Jun-17**	Bf Be Df
**G1_23-Jun-17**	Bd Be Df Ef
**G1_24-Jun-17**	Df Dg Bg
**G4_28-Apr-18**	Bg Bd Be Df Ef Gd
**G4_03-May-18**	Bd Df
**G1_12-May-18**	Gd Ge Gg Gh Ib
**G4_17-May-18**	Bh Bi
**G1_19-May-18**	Gd Gf Gg Ge Bi Bb Bj
**G1_23-May-18**	Bi Bb Ib Bh Bc Gd Gh
**G4_25-May-18**	Gd Gf Gg Bh Bb Ba
**G1_31-May-18**	Gd Ge
**G1_22-Jun-18**	Gd Gg Bh Bi Bb
**G1_01-Jul-18**	Gd Gg Bh Bi Gh Bb Ge Gf Bc Bj Ib
