# Supplementary material for: Song recordings suggest feeding ground sharing in Southern Hemisphere humpback whales
Source: Sci Rep. 2022 Aug 17;12:13924. doi: 10.1038/s41598-022-17999-y (PMC9385655; doi:10.1038/s41598-022-17999-y)
Supplement: Supplementary file 2 — Supplementary Information 2. [file 41598_2022_17999_MOESM2_ESM.pdf]

Song recordings suggest feeding ground sharing in Southern Hemisphere humpback whales

Authors
Elena Schall, Divna Djokic, Erin C. Ross-Marsh, Javier Oña, Judith Denkinger, Julio Ernesto Baumgarten, Linilson Rodrigues Padovese, Marcos R. Rossi-Santos, Maria Isabel Carvalho Goncalves, Renata Sousa-Lima, Rodrigo Hucke-Gaete, Simon Elwen, Susannah Buchan, Tess Gridley, Ilse Van Opzeeland

Humpback whale call and phrase type catalogue – ASSO/Breeding grounds

- ➔ Atlantic sector of the Southern Ocean - **ASSO**
- ➔ Ecuador – **E**
- ➔ Brazil – **B**
- ➔ South Africa – **A**

- Call Types –

Representation of the call types used as song units.

Name	Example	Description
CT1<br>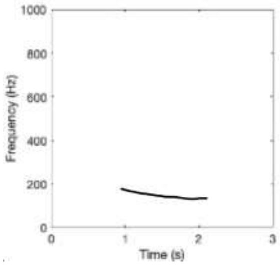	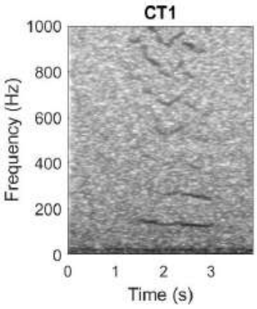	- tonal<br>- Fundamental 100-300Hz<br>- 1-2s<br>- Constant frequency contour<br>- Slightly downswept
CT3<br>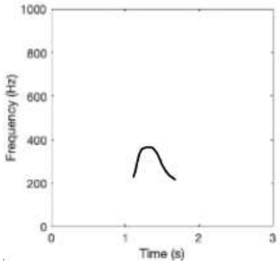	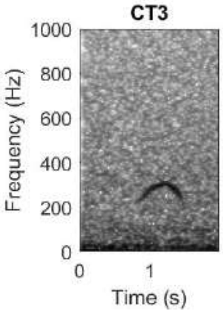	- tonal<br>- Fundamental 100-300Hz<br>- 0.5-1s<br>- Up-downsweep<br>- Sometimes up- or downsweep dominates
CT4a<br>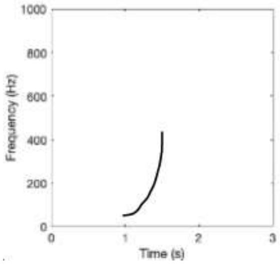	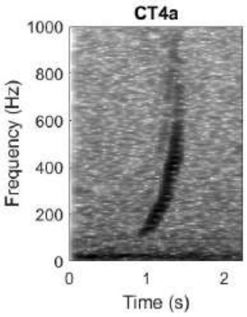	- tonal<br>- Fundamental (a) 80-800Hz<br>- Fundamental (b) 500-1200Hz<br>- 0.3-0.7s<br>- Upsweep<br>- Sometimes almost pulsed

CT5b 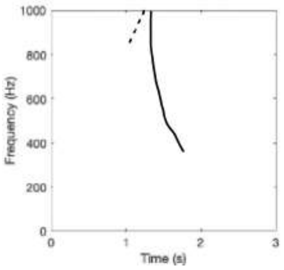	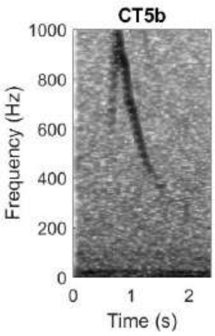	- tonal<br>- Fundamental (a)100-500Hz<br>- Fundamental (b)300-1200Hz<br>- 0.3-1.2s<br>- Downsweep<br>- Sometimes up-downsweep, sometimes upsweep, sometimes almost pulsed
CT6 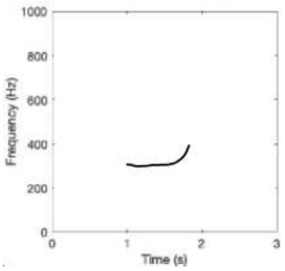	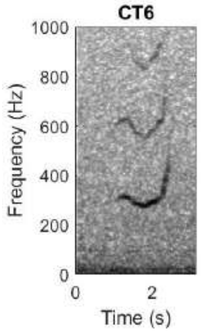	- tonal<br>- Fundamental 200-600Hz<br>- 1-2s<br>- Constant-upsweep<br>- Sometimes almost upsweep
CT7 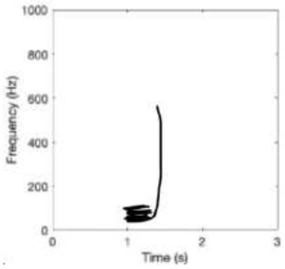	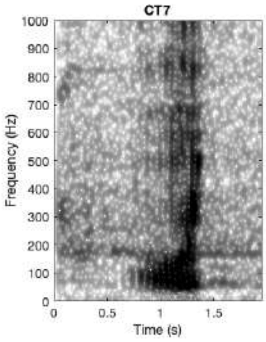	- Pulsed & tonal<br>- 40-1500Hz<br>- 0.3-0.8s<br>- Amplitude modulated<br>- Final upsweep as CT4a
CT8 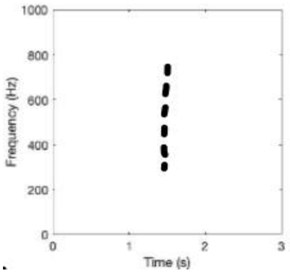	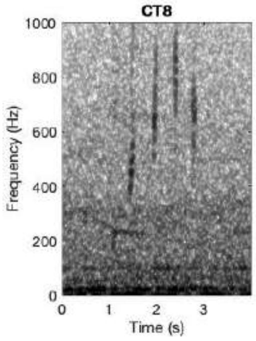	- Pulsed<br>- 300->2500Hz<br>- 0.1-0.3s<br>- Amplitude modulated<br>- Sometimes almost upsweep
CT10 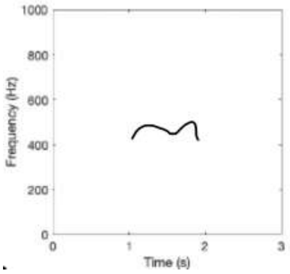	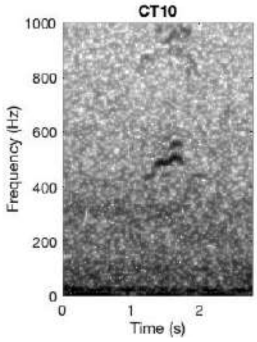	- tonal<br>- Fundamental 400-600Hz<br>- 0.5-2s<br>- Irregular frequency modulations<br>- Sometimes almost broadband

CT12<br>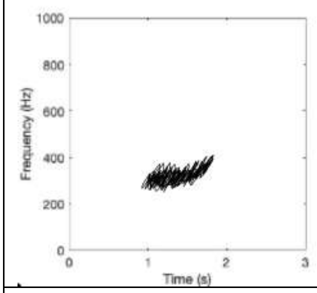	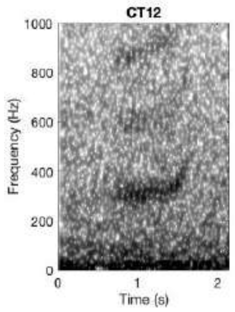	- Pulsed<br>- 200-500Hz<br>- 0.5-2s<br>- Broadband with increasing frequency<br>- Sometimes with 'harmonics' (Amplitude modulated)
CT13b<br>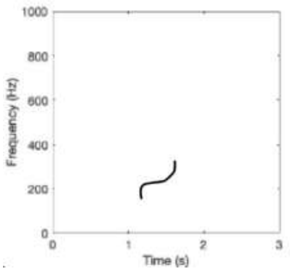	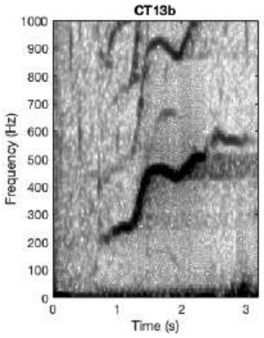	- Tonal<br>- Fundamental (a) 50-300Hz<br>- Fundamental (b) 200-700Hz<br>- Fundamental (c) 500-1500Hz<br>- 0.5-2s<br>- Stepwise increase in frequency
CT15<br>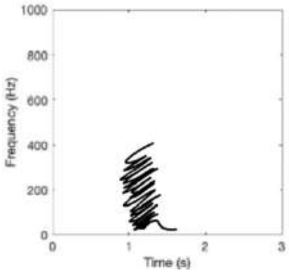	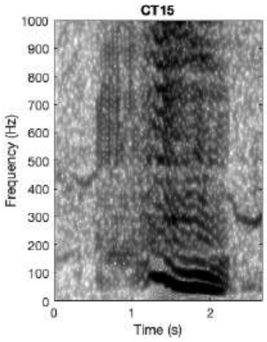	- Pulsed & tonal<br>- 40-1500Hz<br>- 1-3s<br>- Amplitude and frequency modulated<br>- Final downsweep
CT16<br>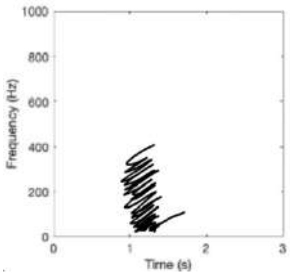	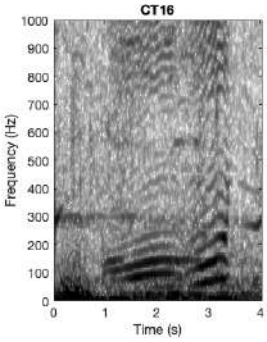	- Pulsed & tonal<br>- 40-1500Hz<br>- 1-3s<br>- Amplitude and frequency modulated<br>- Final upsweep
CT17<br>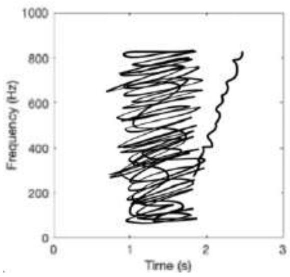	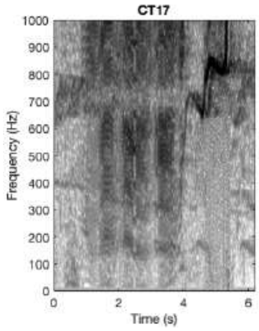	- Pulsed & tonal<br>- 40-1500Hz<br>- 2-5s<br>- Amplitude and frequency modulated<br>- Final high frequency upsweep

CT18 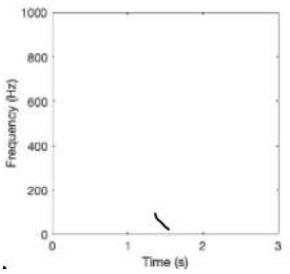	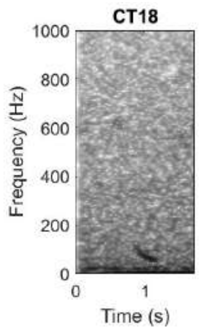	- tonal<br>- Fundamental 20-120Hz<br>- 0.2-1s<br>- Downsweep<br>- Sometimes up-downsweep
CT19 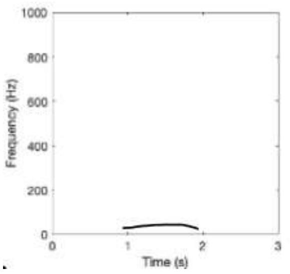	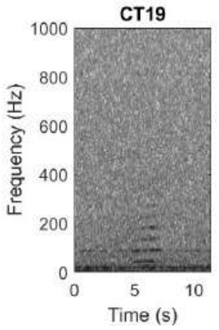	- tonal<br>- Fundamental 20-100Hz<br>- 1-4s<br>- Constant frequency<br>- Sometimes rather up-downsweep, down-upsweep, downsweep or upsweep

- ➔ Each phrase type is characterized by a distinct combination of units.
- ➔ Each phrase type is named by a capital letter and a lowercase letter, where the capital letter is an indicator for the first unit of the phrase type: i.e., Aa and Ab both start with call type (CT) 1.
- ➔ A phrase type is divided into phrase subtypes, when the number of repetitions of respective units differs: i.e., 1x CT1 and 3x CT4 translate into Aa13.

Type Aa

The combination of units CT1 & CT4a

<table>
<tr><th colspan="2">Example of Aa13</th></tr>
<tr><td>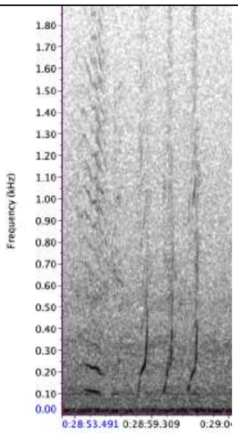
</td><td>ASSO, B
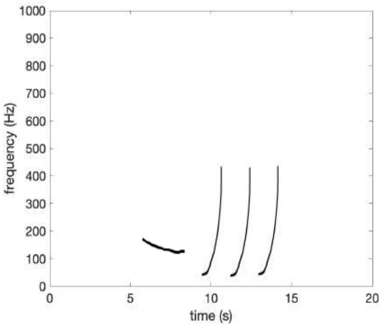
</td></tr>
</table>

Type Ab

Combination CT1, CT4a & CT5b

Example of Ab123	
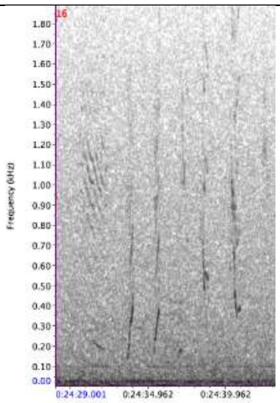	**ASSO** 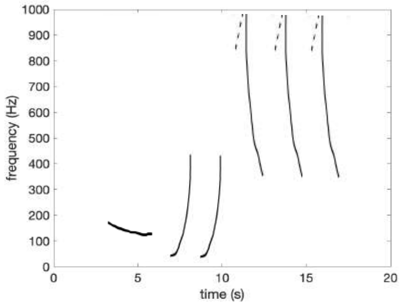

Type Ac

Combination of units CT1 & CT5b

Example of Ac13	
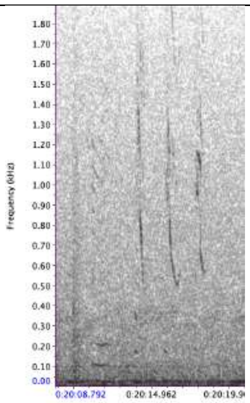	**ASSO** 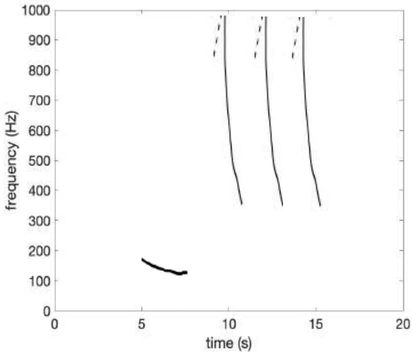

Type Ad

Combination of units CT1, CT5b, CT4a

Example of Ad123

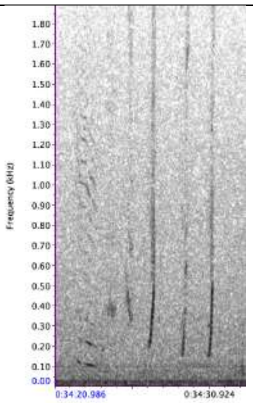

ASSO

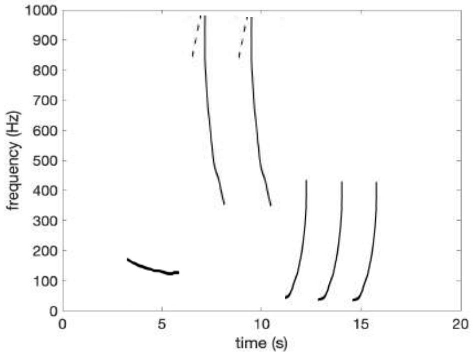

Type Ae

Combination of units CT1, CT6, CT5b

Example of Ae114

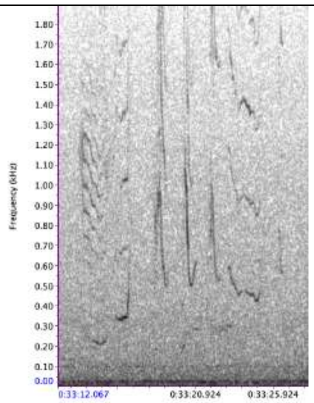

ASSO

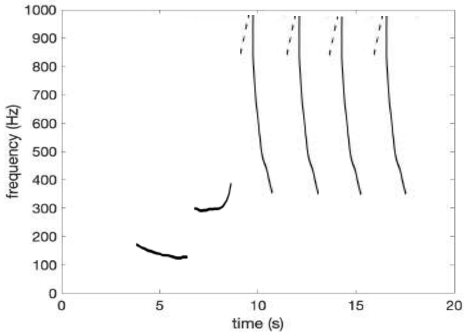

Type Af

Combination of units CT1, CT12, CT18

Example of Af112	
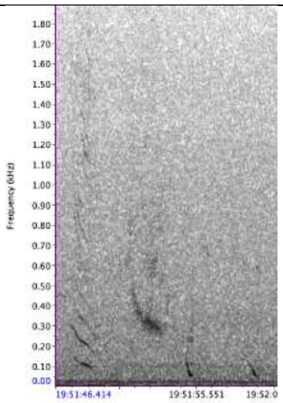	**ASSO, B** 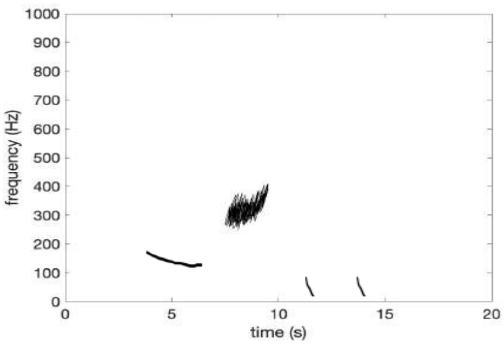

Type Ag

Combination of units CT1 and CT18

Example of Ag11	
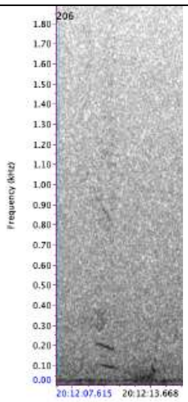	**ASSO** 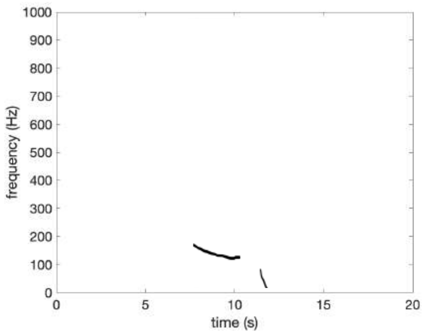

Type Ah

Combination of units CT1, CT12, CT4a

<table>
<tr><th colspan="2">Example of Ah112</th></tr>
<tr><td>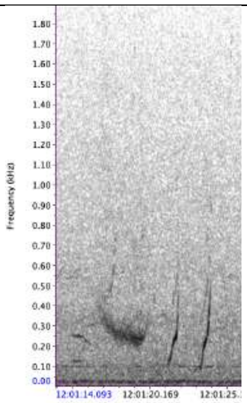
</td><td>**ASSO**

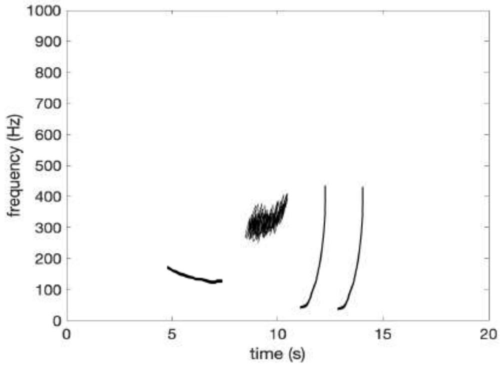
</td></tr>
</table>

Type Ai

Combination of units CT1 and CT12

<table>
<tr><th colspan="2">Example of Ai11</th></tr>
<tr><td>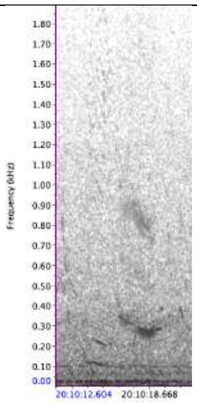
</td><td>**ASSO**

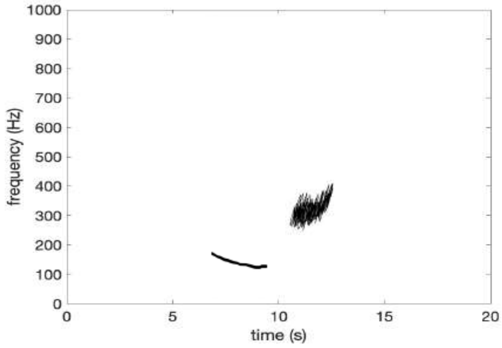
</td></tr>
</table>

Type Aj

Combination of units CT1, CT12, CT5b

<table>
<tr><td colspan="2">Example of Af114</td></tr>
<tr><td>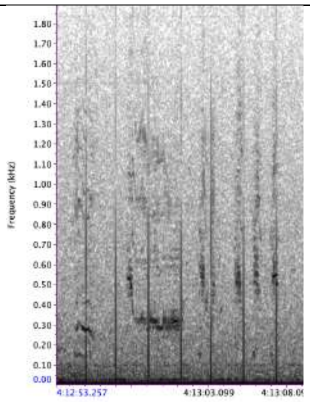
</td><td>ASSO
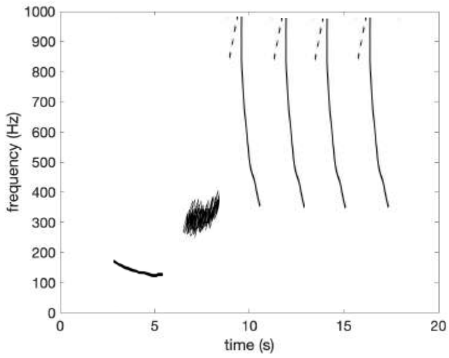
</td></tr>
</table>

Type Ak

Combination of units CT1 & CT8

<table>
<tr><td colspan="2">Example of Ak120</td></tr>
<tr><td>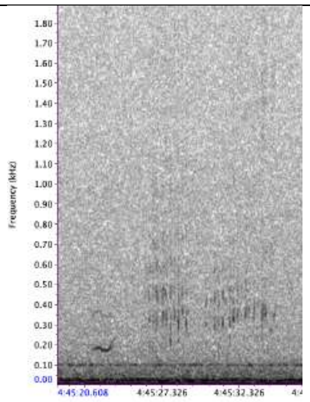
</td><td>ASSO
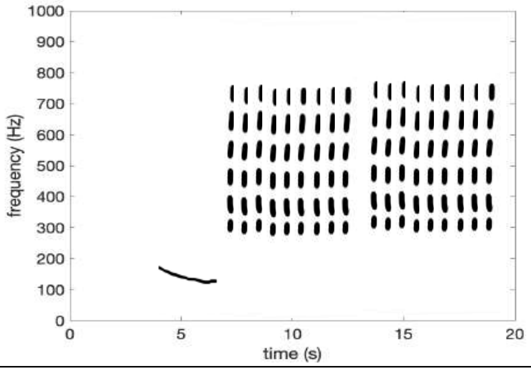
</td></tr>
</table>

Type Al

Combination of units CT1, CT8, CT4a

Example of Al1171

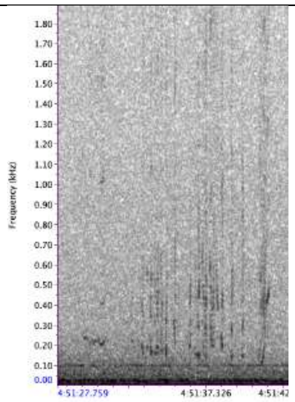

ASSO

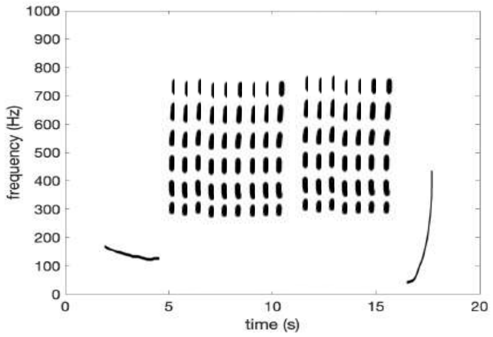

Type Am

Combination of units CT1, CT4a, CT12

Example of Am122

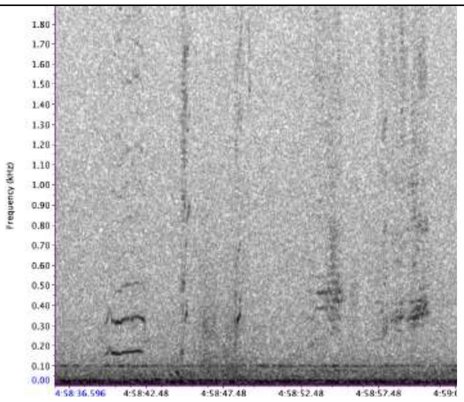

ASSO

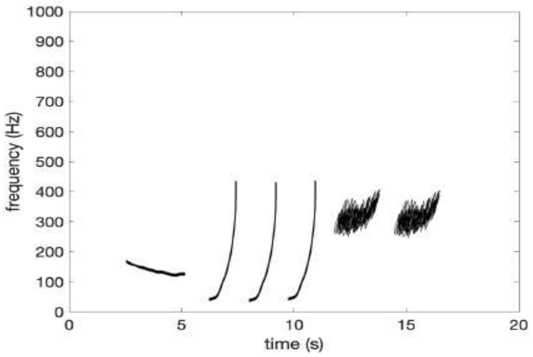

Type An

Combination of units CT1, CT12, CT8

Example of An1116	
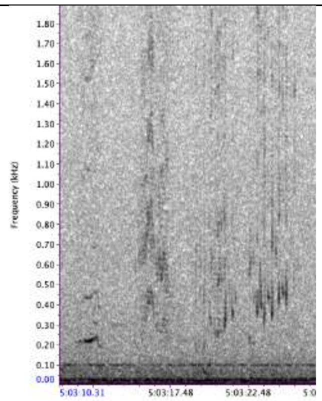	**ASSO** 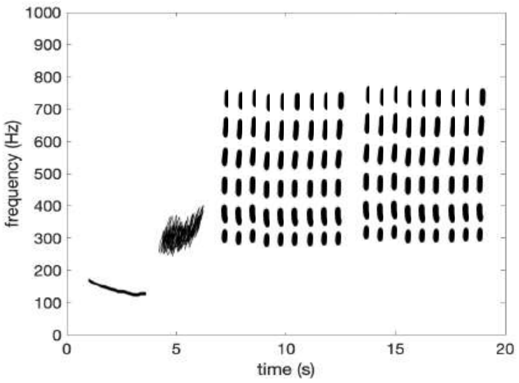

Type Ao

Combination of units CT1, CT5b/a & CT12

Example of Ao111	
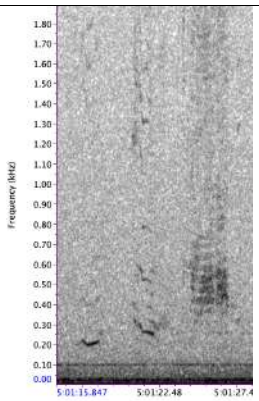	**ASSO** 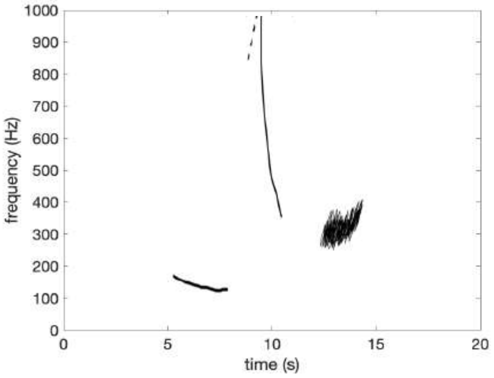

Type Ap

Combination of units CT1, CT4a & CT18

<table>
<tr><th colspan="2">Example of Ap11214</th></tr>
<tr><td>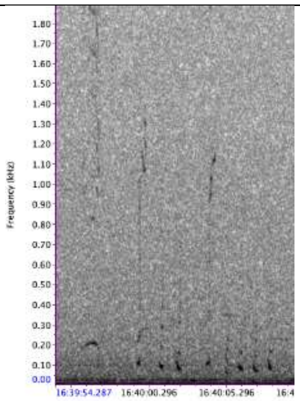
</td><td>ASSO
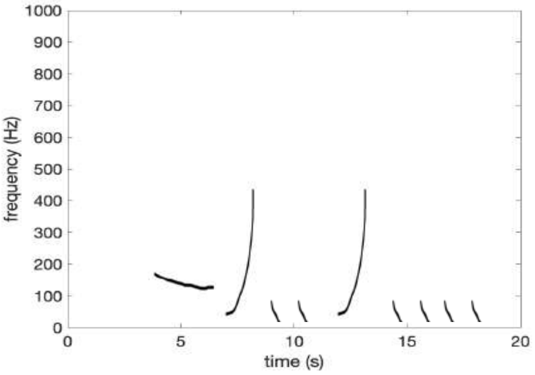
</td></tr>
</table>

Type Aq

Combination of units CT1, CT5b & CT18

<table>
<tr><th colspan="2">Example of Aq112121211</th></tr>
<tr><td>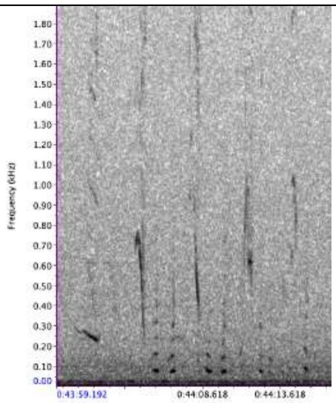
</td><td>ASSO
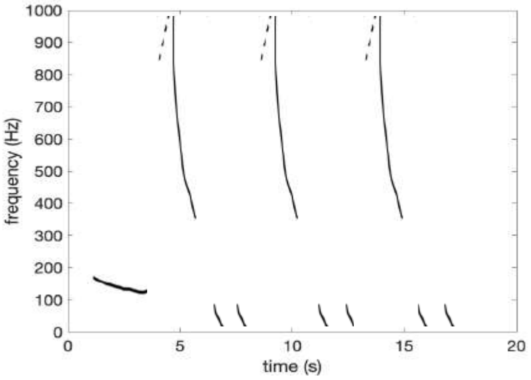
</td></tr>
</table>

Type Ar

Combination of units CT1, CT10

Example of Ar11	
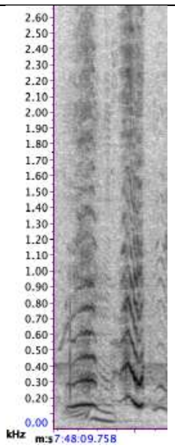	**B, E** 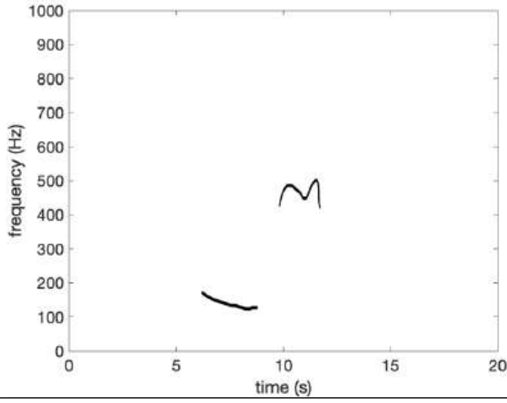

Type As

Combination of units CT1, CT15

Example of As11	
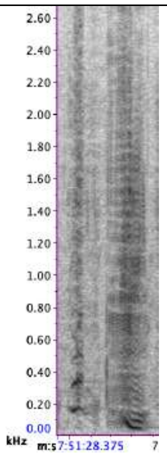	**B, E** 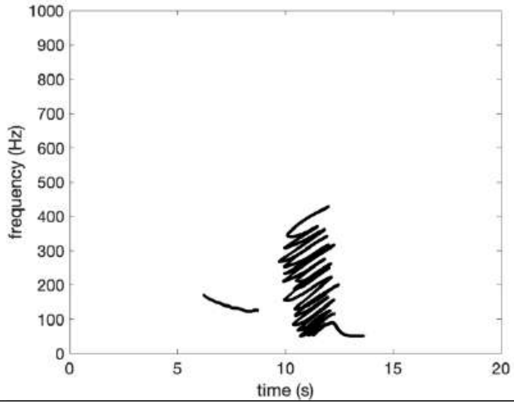

Type At

Combination of units CT1, CT5a

Example of At12	
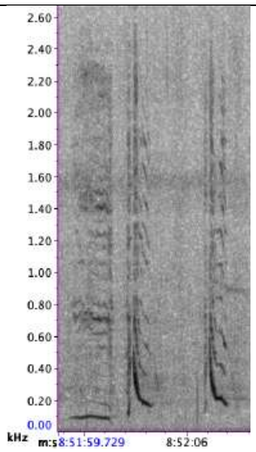	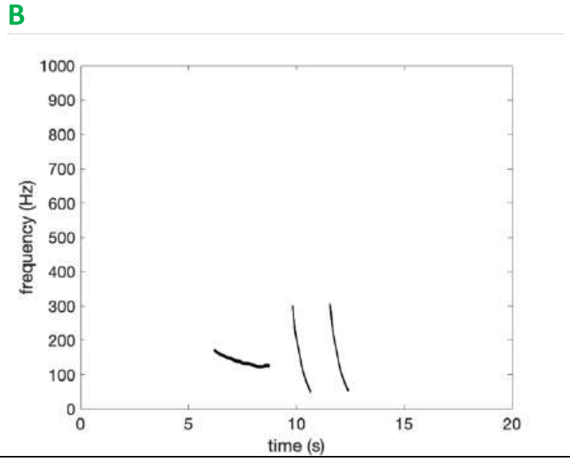

Type Au

Combination of units CT1, CT15, CT4a, CT19

Example of Au1112	
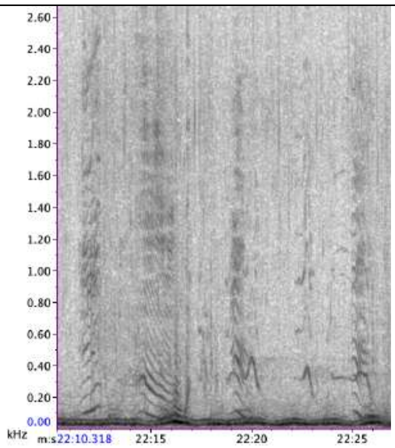	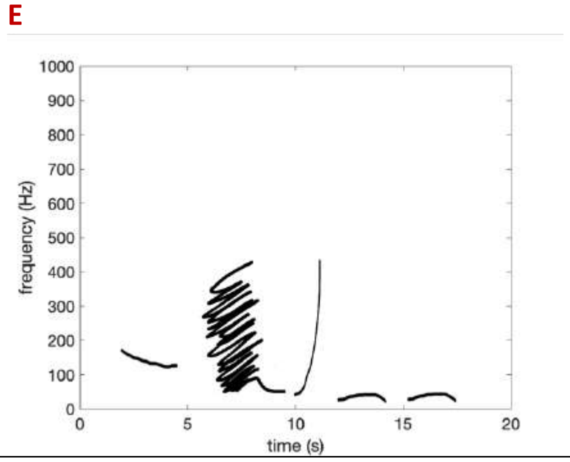

Type Av

Combination of units CT1, CT16, CT10

Example of Av211	
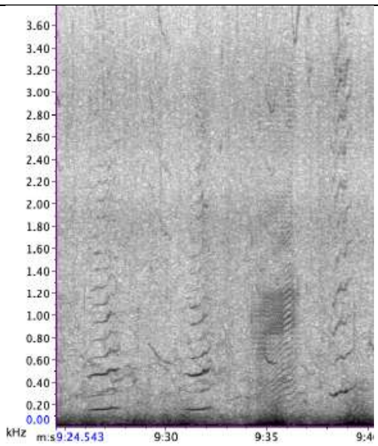	E<br>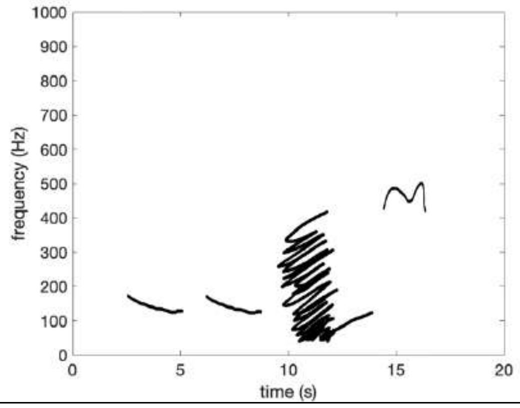

Type Aw

Combination of units CT1, CT13b

Example of Aw31	
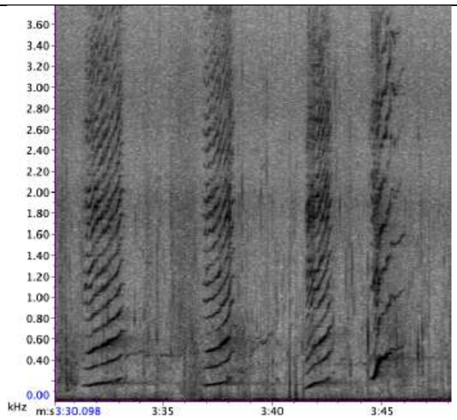	E<br>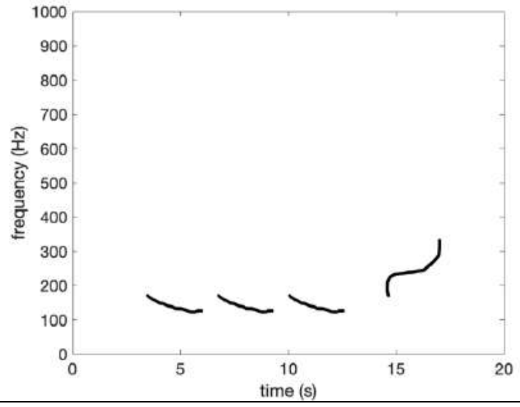

Type Ax

Combination of units CT1, CT16, CT13b

Example of Ax211	
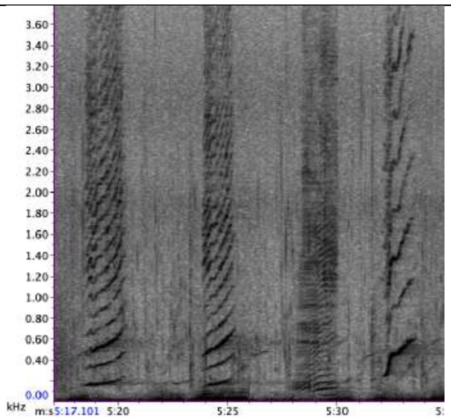	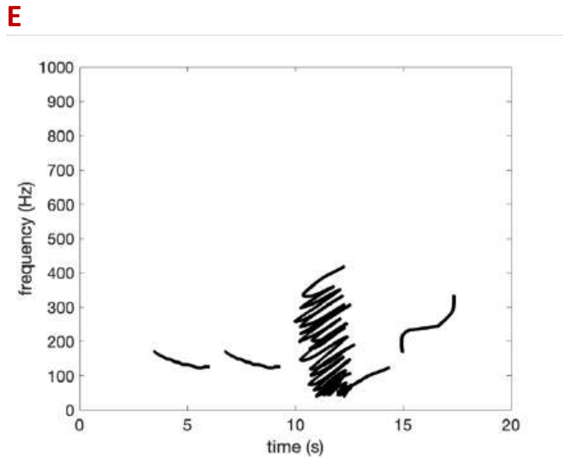

Type Ay

Combination of units CT1, CT15, CT10

Example of Ay211	
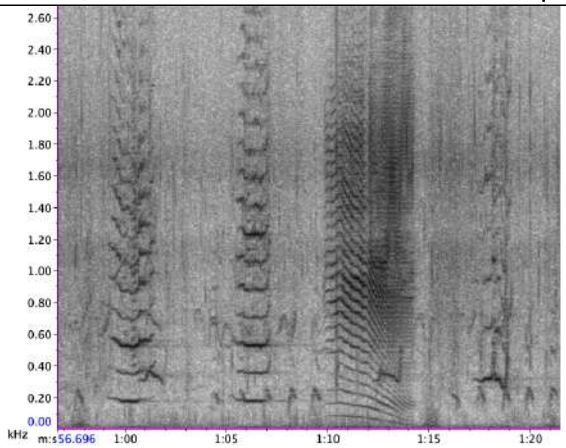	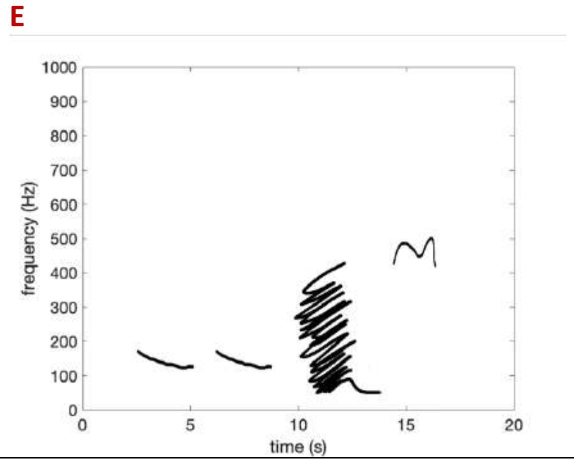

Type Ba

The combination of units CT6 & CT5b

<table>
<tr><th colspan="2">Example of Ba17</th></tr>
<tr><td>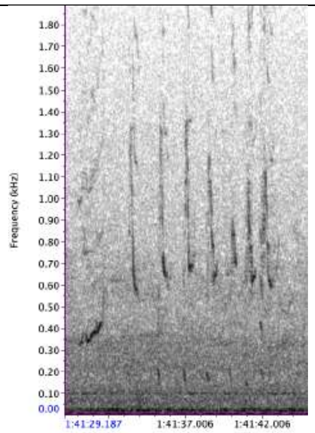
</td><td>ASSO, B

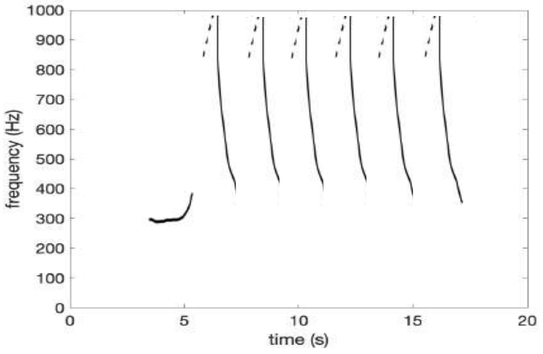
</td></tr>
</table>

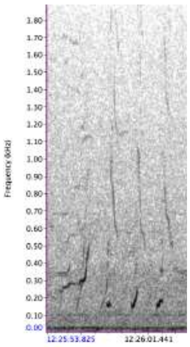

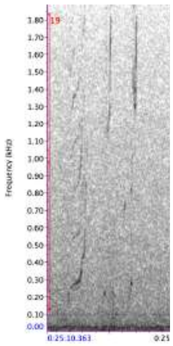

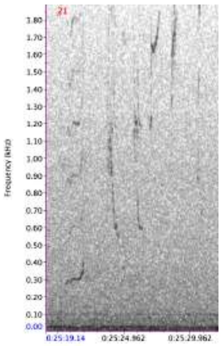

Type Bb

Combination of units CT6 & CT4a

<table>
<tr><th colspan="2">Example of Bb12</th></tr>
<tr><td>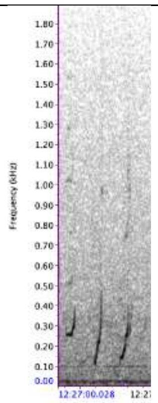
</td><td>ASSO, B, A

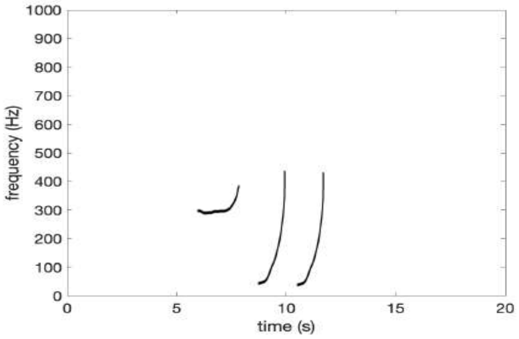
</td></tr>
</table>

Type Bc

Combination of units CT6 & CT12

Example of Bc12

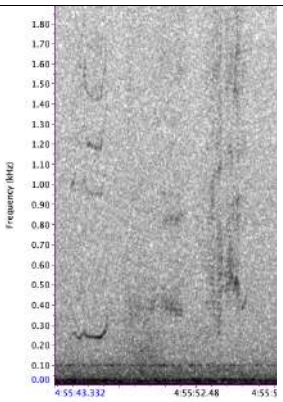

ASSO, B, A

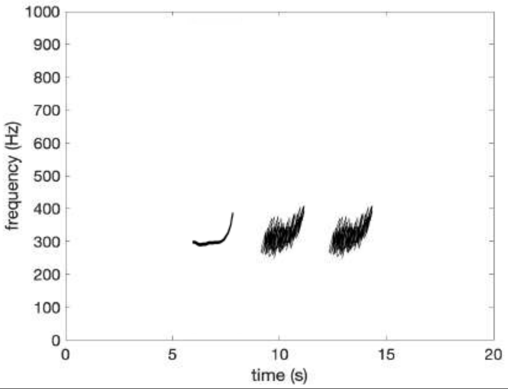

Type Bd

Combination of units CT6, CT4a, CT10 & CT4b

Example of Bd2111

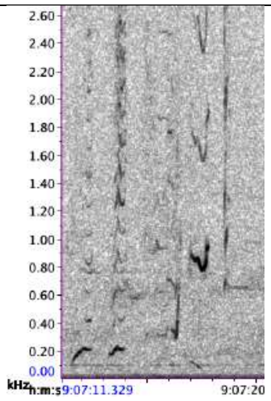

ASSO

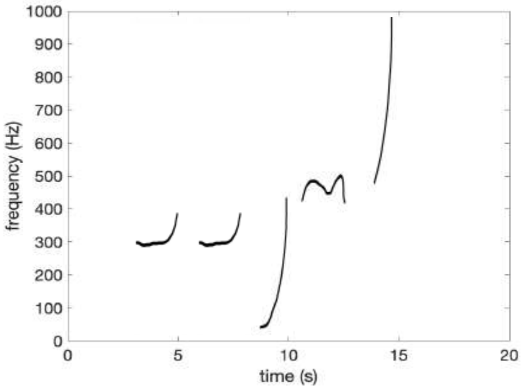

Type Be

Combination of units CT6, CT10

<table>
<tr><th colspan="2">Example of Be11</th></tr>
<tr><td>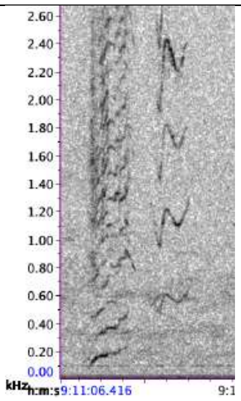
</td>
<td>ASSO, B
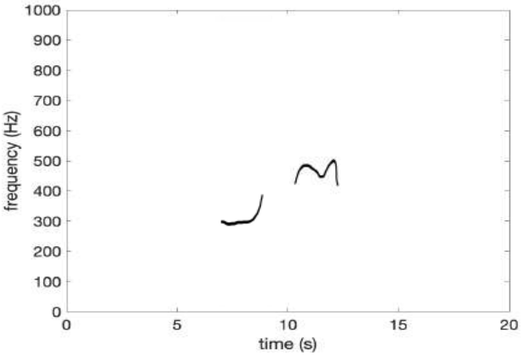
</td></tr>
</table>

Type Bf

Combination of units CT6, CT4a, CT10

<table>
<tr><th colspan="2">Example of Bf211</th></tr>
<tr><td>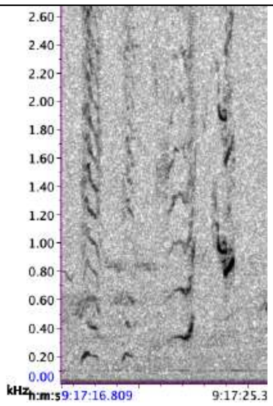
</td>
<td>ASSO
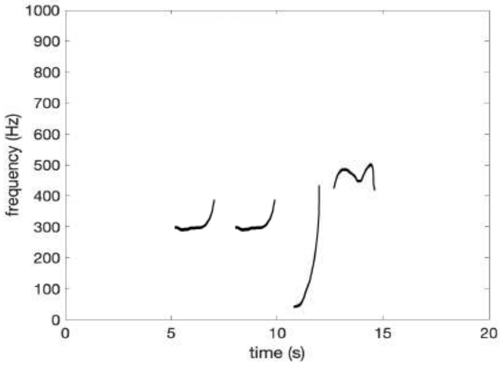
</td></tr>
</table>

Type Bg

Combination of units CT6, CT10, CT4b

<table>
<tr><td colspan="2">Example of Bg111</td></tr>
<tr><td>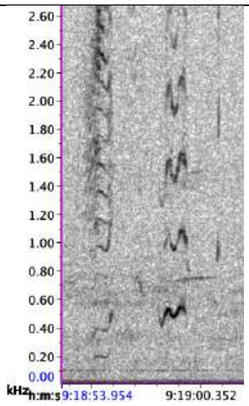
</td><td>ASSO
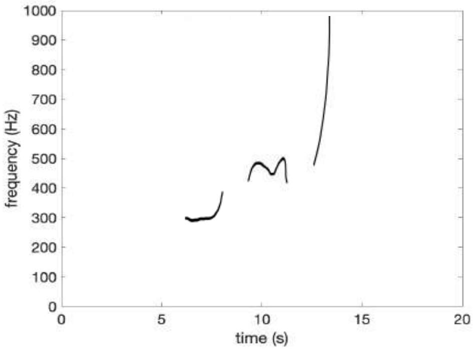
</td></tr>
</table>

Type Bh

Combination of units CT6, CT1

<table>
<tr><td colspan="2">Example of Bh13</td></tr>
<tr><td>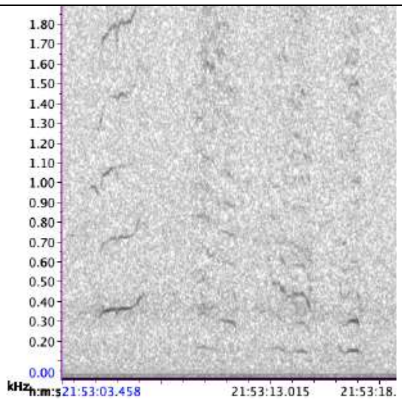
</td><td>ASSO, B, A
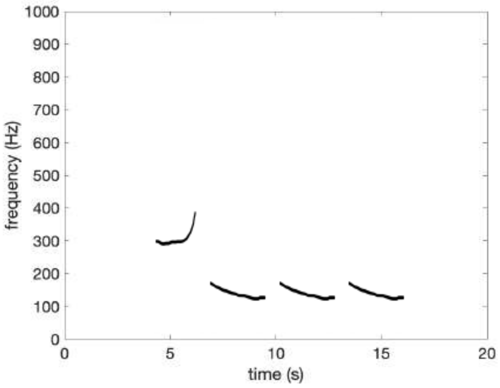
</td></tr>
</table>

Type Bi

Combination of units CT6, CT1, CT4a

Example of Bi143

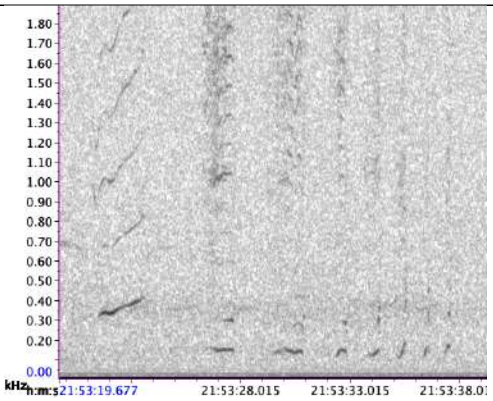

ASSO, B, A

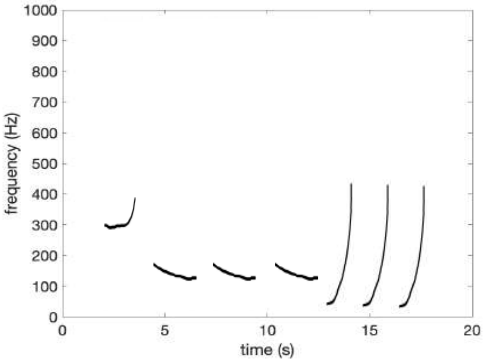

Type Bj

Combination of units CT6, CT12, CT4a

Example of Bj123

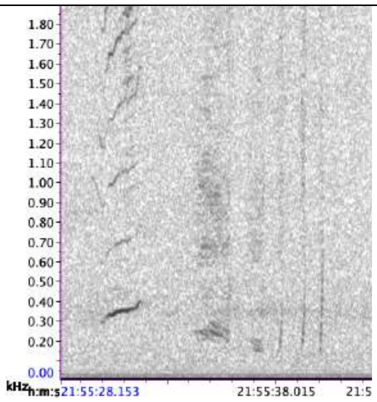

ASSO, A

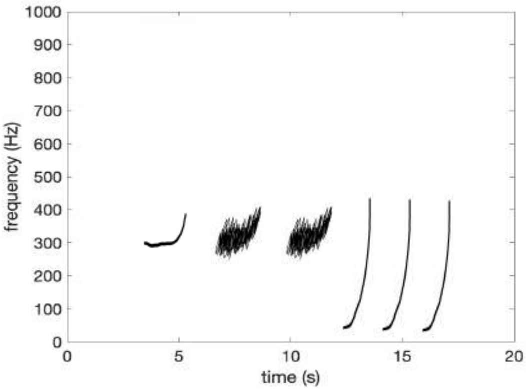

Type Bk

Combination of units CT6, CT18

<table>
<tr><th colspan="2">Example of Bk15</th></tr>
<tr><td>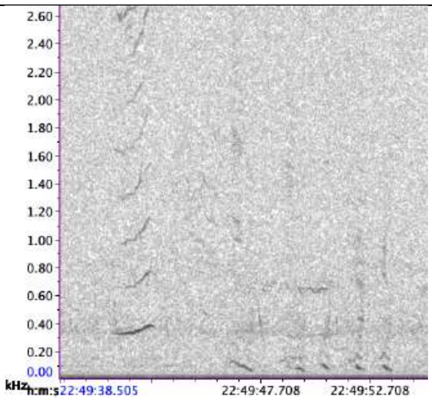
</td>
<td>ASSO, B, A
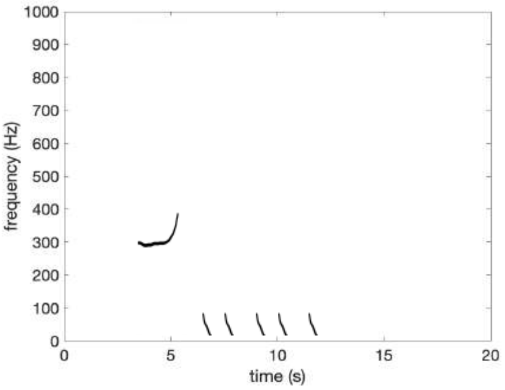
</td></tr>
</table>

Type Bl

Combination of units CT6, CT5a, CT10

<table>
<tr><th colspan="2">Example of Bk15</th></tr>
<tr><td>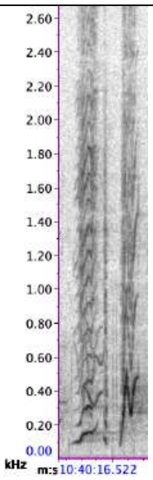
</td>
<td>B
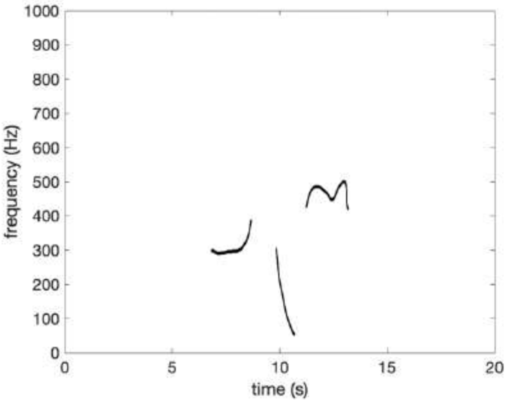
</td></tr>
</table>

Type Bm

Combination of units CT6, CT7

Example of Bm13	
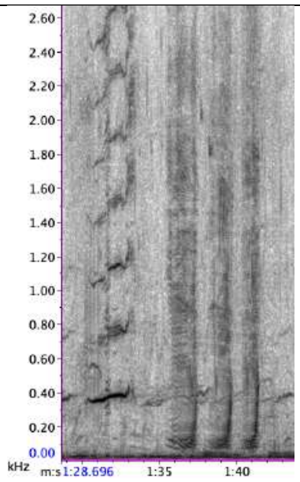	**B, A** 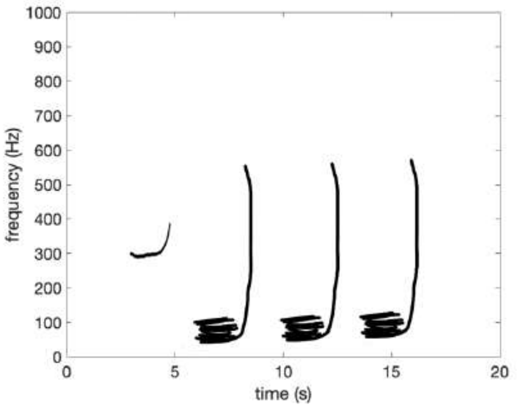

Type Bn

Combination of units CT6, CT19

Example of Bn12	
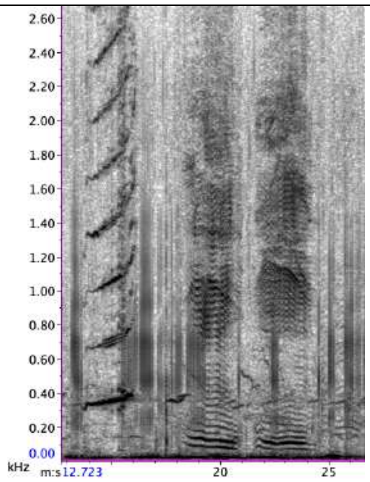	**B** 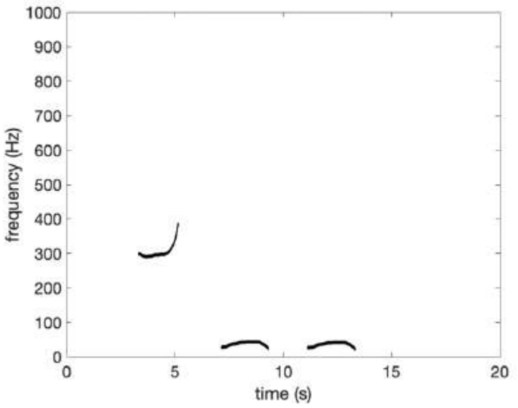

Type Bo

Combination of units CT6, CT8

Example of Bo14

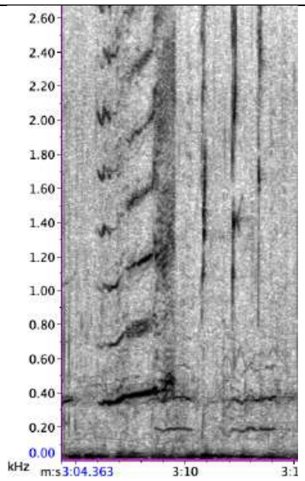

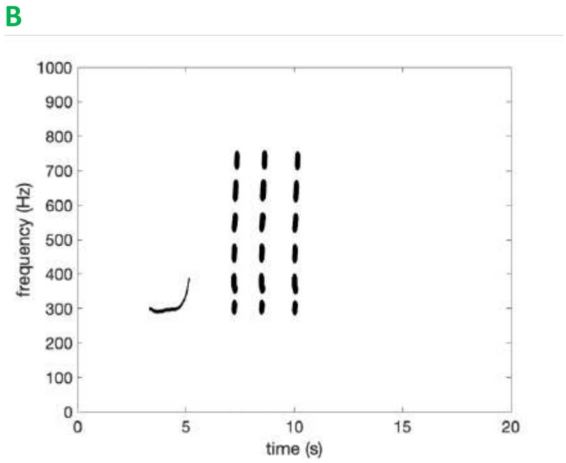

Type Bp

Combination of units CT6, CT8, CT10

Example of Bp171

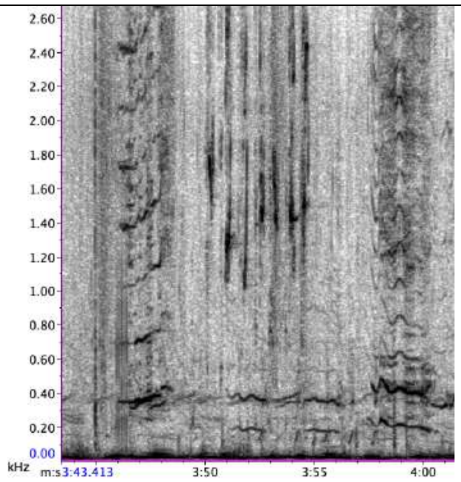

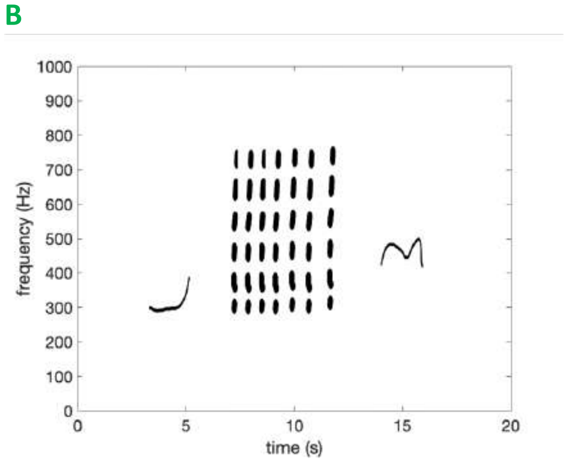

Type Bq

Combination of units CT6, CT4a, CT10, CT5b

<table>
<tr><th colspan="2">Example of Bq1111</th></tr>
<tr><td>
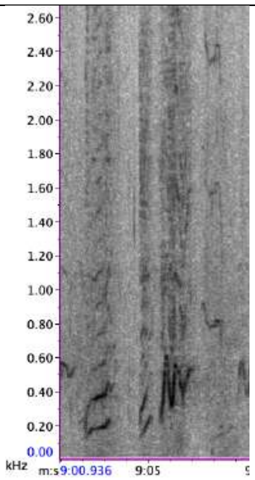

</td><td>
B
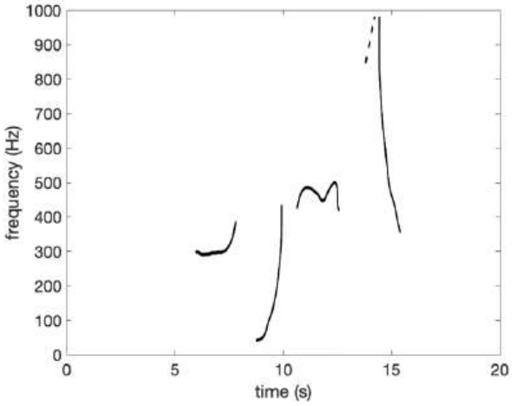

</td></tr>
</table>

Type Ca

Combination of units CT3 & CT5b

<table>
<tr><th colspan="2">Example of Ca13</th></tr>
<tr><td>
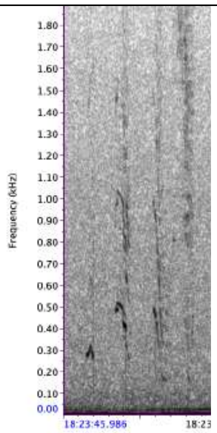

</td><td>
ASSO
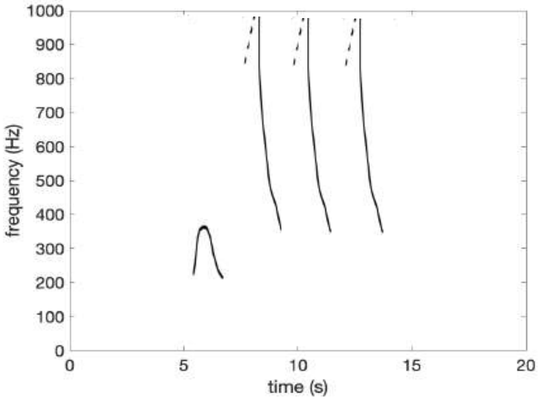

</td></tr>
</table>

Type Cb

Combination of units CT3, CT18

Example of Cb11	
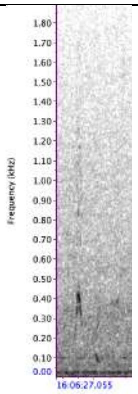	**ASSO** 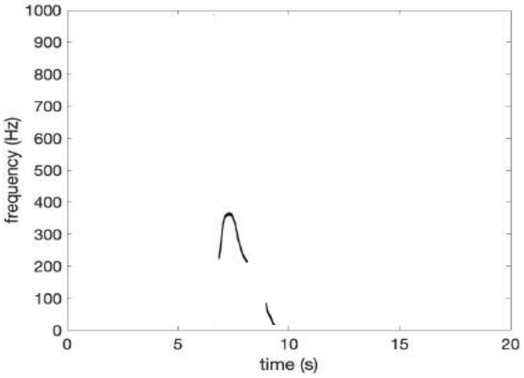

Type Cc

Combination of units CT3 and CT10

Example of Cc12	
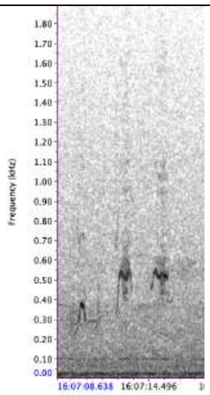	**ASSO** 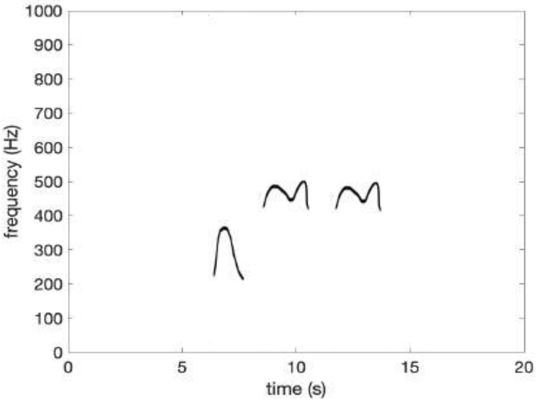

Type Cd

Combination of units CT3, CT18, CT10

Example of Cd111	
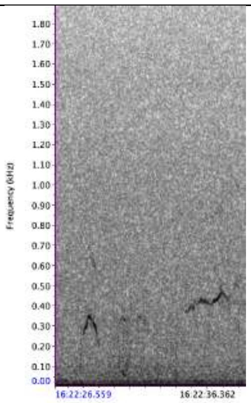	**ASSO** 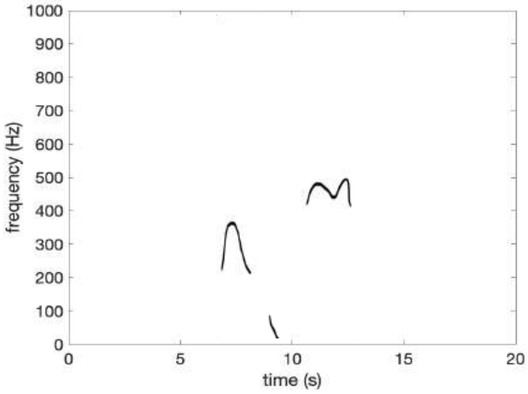

Type Ce

Combination of units CT3, CT10, CT18

Example of Ce111	
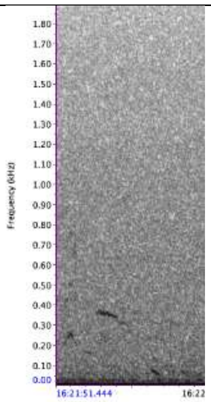	**ASSO** 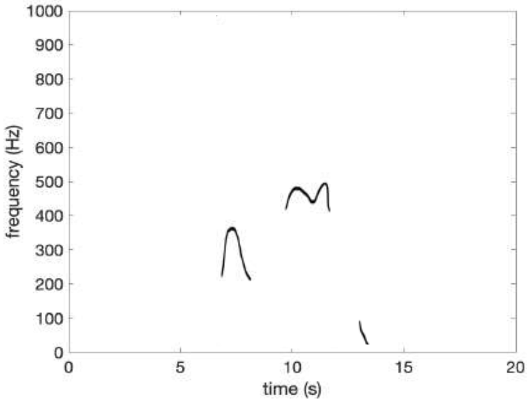

Type Cf

Combination of units CT3, CT10, CT5b

Example of Cf115	
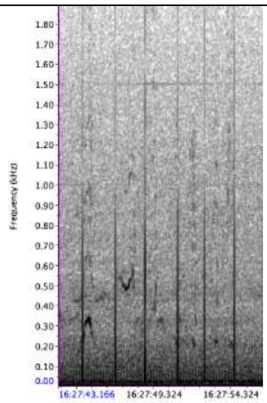	**ASSO** 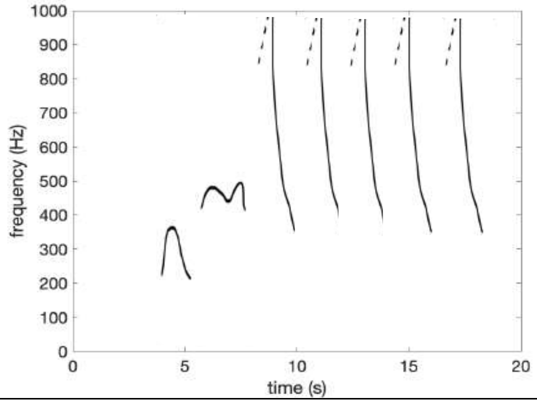

Type Cg

Combination of units CT3, CT10

Example of Cg32	
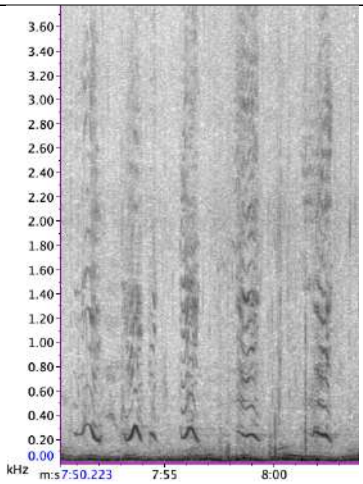	**E** 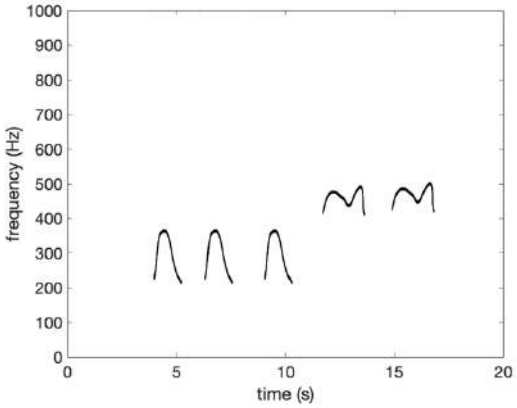

Type Da

Combination of units CT12 and CT18

Example of Da12	
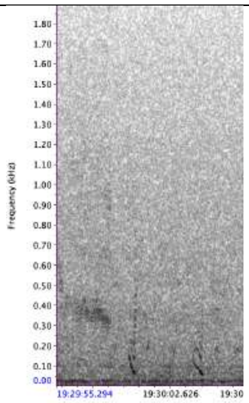	ASSO, B 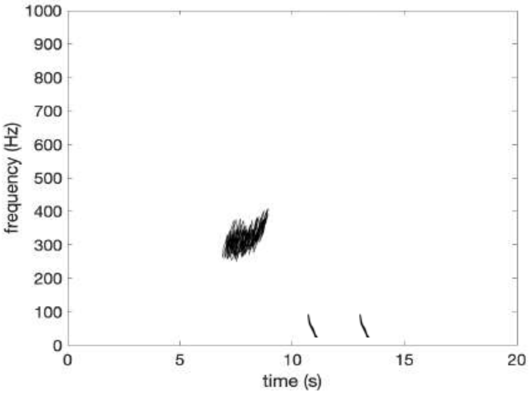

Type Db

Combination of units CT12 and CT5b

Example of Db13	
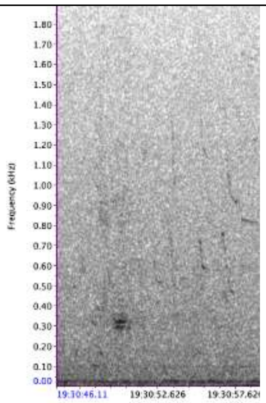	ASSO, B 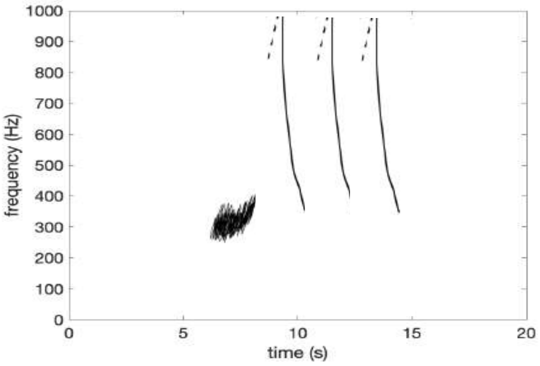

Type Dc

Combination of units CT12, CT4a

Example of Dc13	
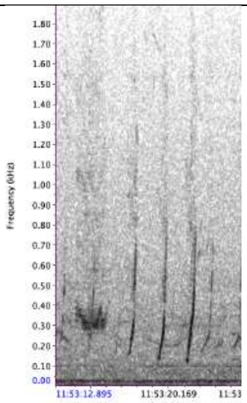	**ASSO**<br>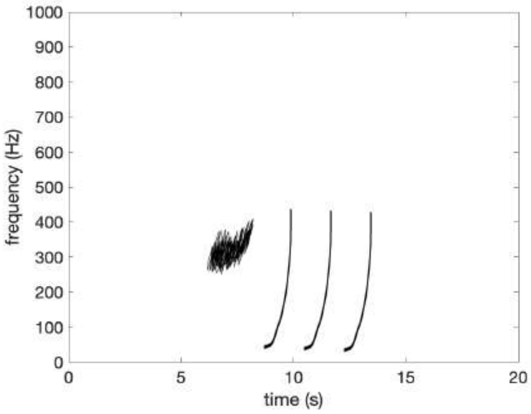

Type Dd

Combination of units CT12, CT1, CT4a

Example of Dd112	
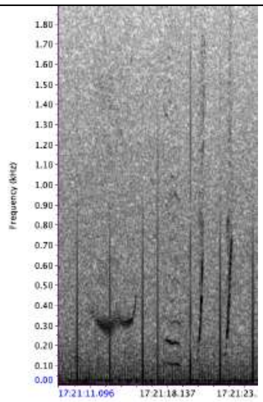	**ASSO**<br>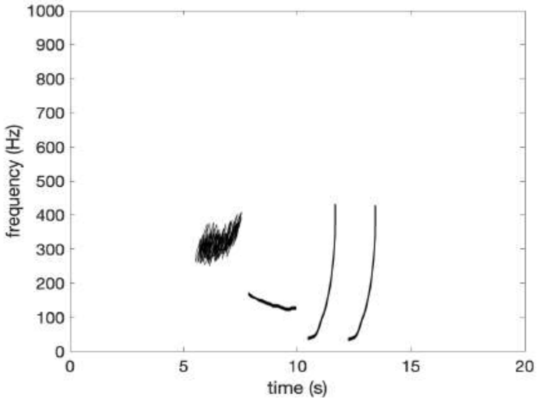

Type De

Combination of units CT12, CT18, CT5b

<table>
<tr><th colspan="2">Example of De116</th></tr>
<tr><td>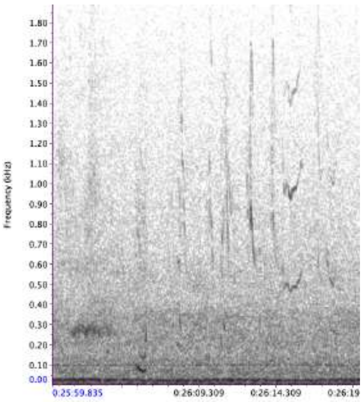
</td>
<td>ASSO
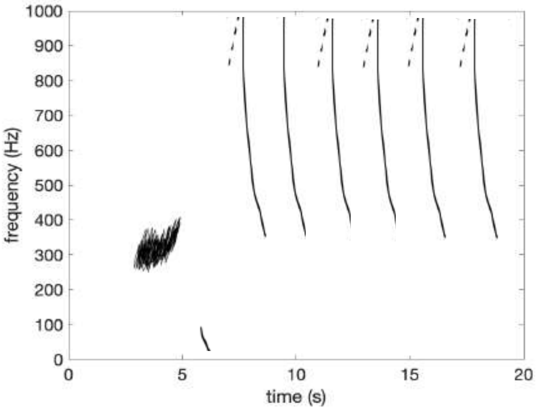
</td></tr>
</table>

Type Df

Combination of units CT12, CT10

<table>
<tr><th colspan="2">Example of Df11</th></tr>
<tr><td>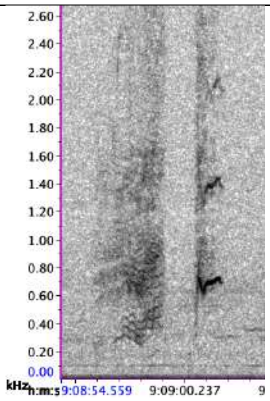
</td>
<td>ASSO, B
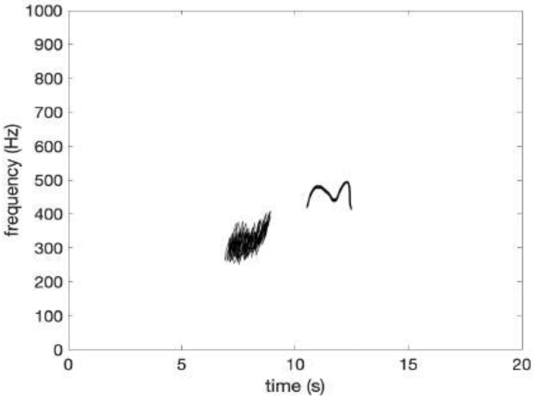
</td></tr>
</table>

Type Dg

Combination of units CT12, CT10, CT4b

<table>
<tr><th colspan="2">Example of Dg111</th></tr>
<tr><td>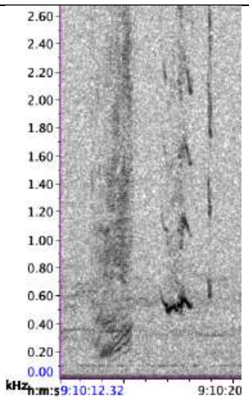
</td><td>ASSO, E
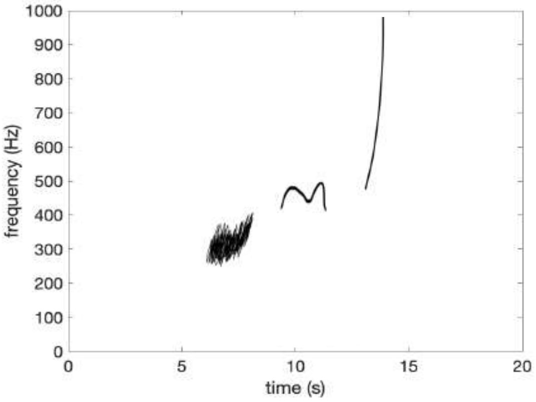
</td></tr>
</table>

Type Dh

Combination of units CT12, CT10, CT4b, CT4a

<table>
<tr><th colspan="2">Example of Dh1111</th></tr>
<tr><td>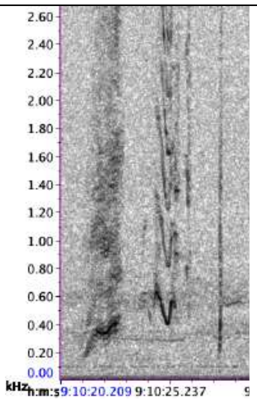
</td><td>ASSO
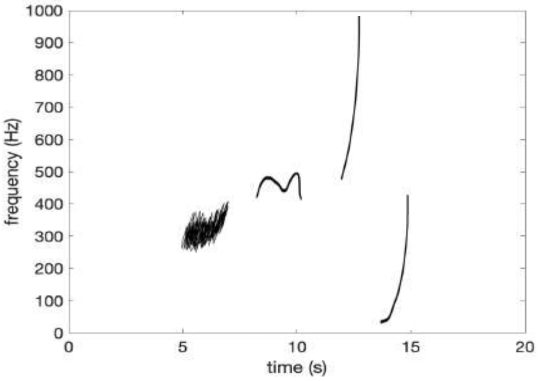
</td></tr>
</table>

Type Di

Combination of units CT12, CT5b/a, CT8

Example of Di1115

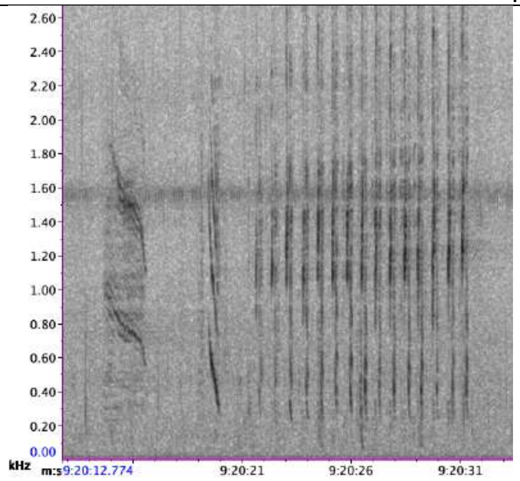

B

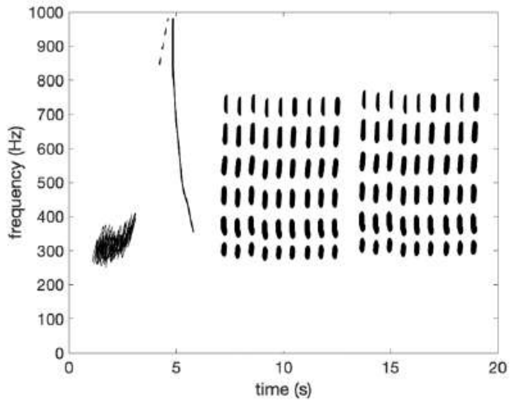

Type Dj

Combination of units CT12, CT19, CT4a

Example of Dj111111111112

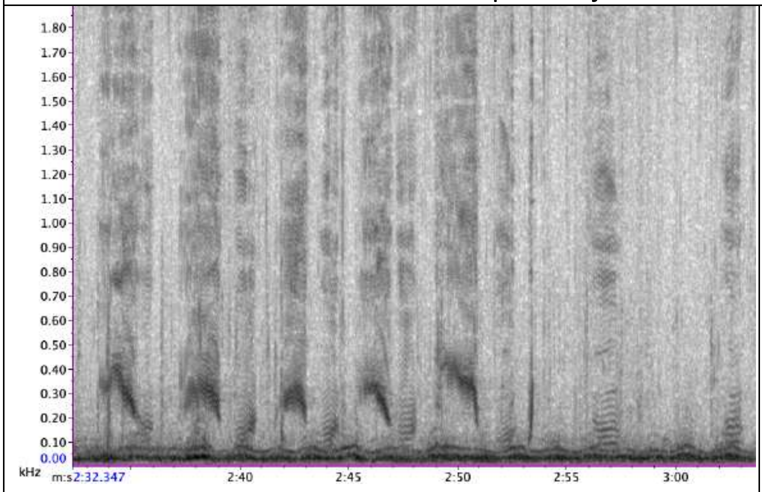

E, B

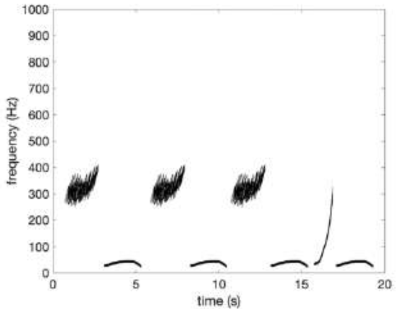

Type Dk

Combination of units CT12, CT8

Example of Dk314	
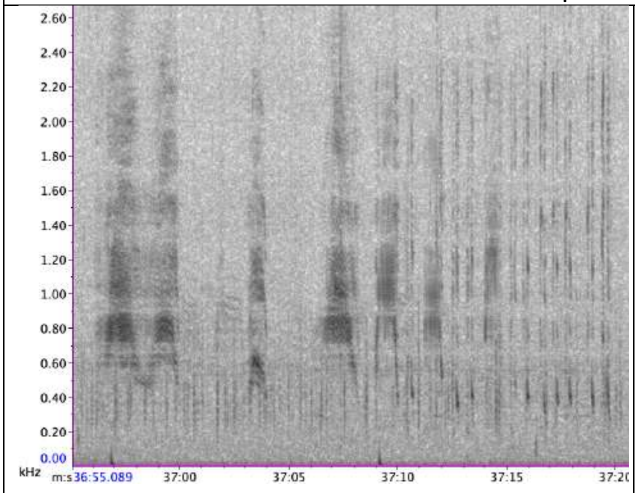	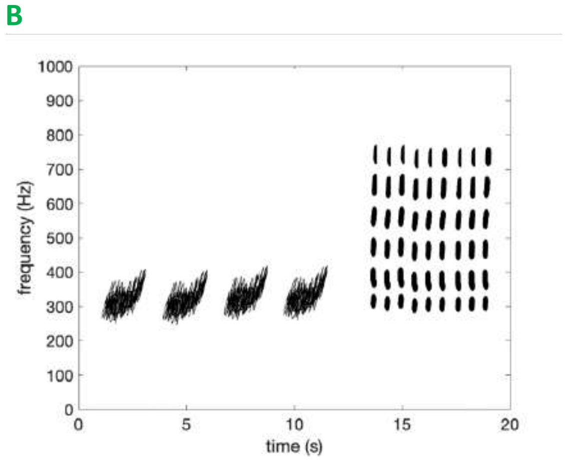

Type Dl

Combination of units CT12, CT7, CT18

Example of Dl12111	
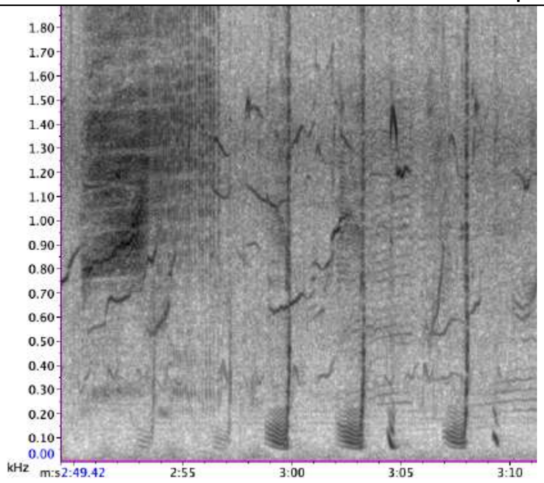	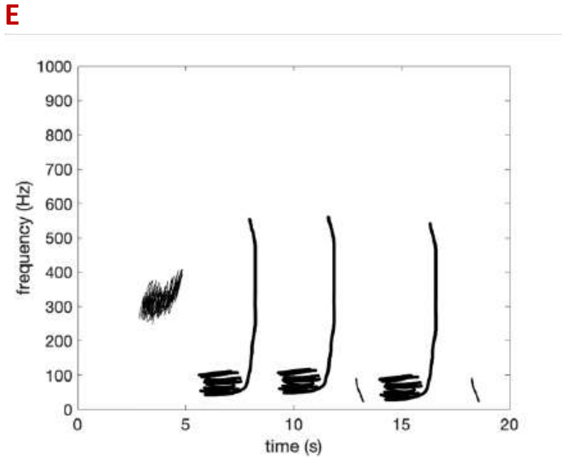

Type Dm

Combination of units CT12, CT13c

Example of Dm12	
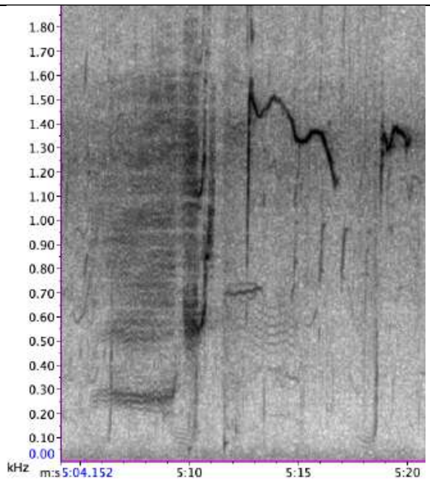	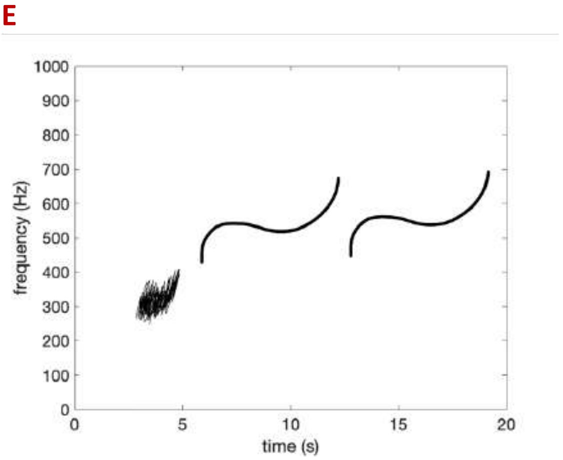

Type Dn

Combination of units CT12, CT8, CT10

Example of Dn141	
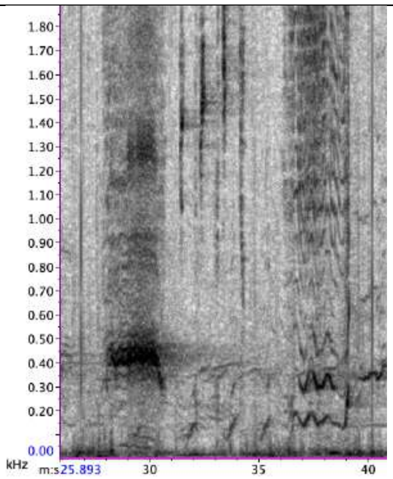	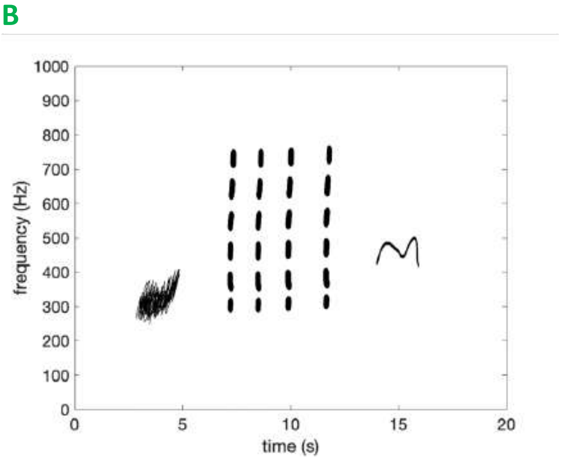

Type Do

Combination of units CT12, CT5a, CT4a

Example of Do121	
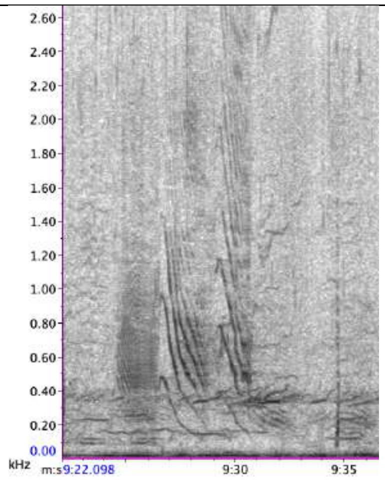	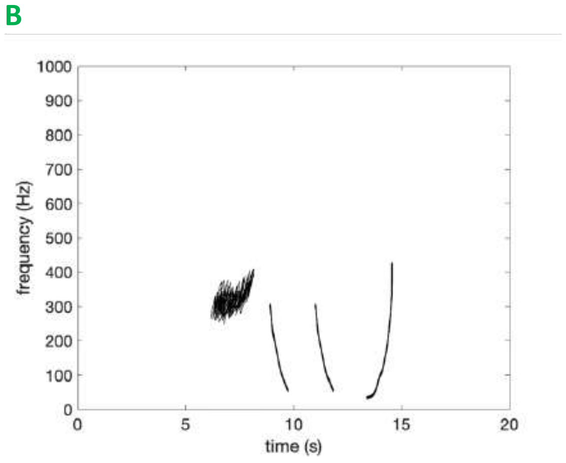

Type Ea

Combination of units CT4a, CT8

Example of Ea13	
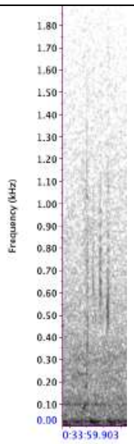	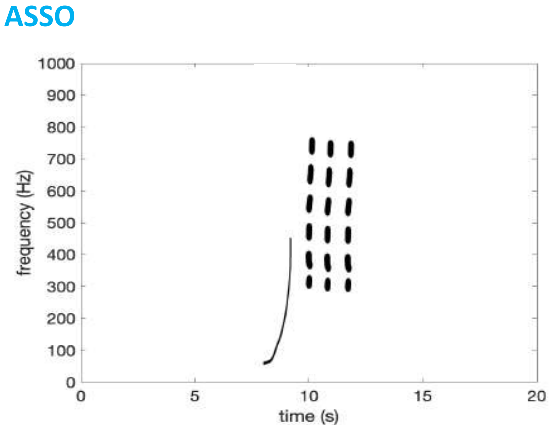

Type Eb

Combination of units CT4a, CT5b

Example of Eb28

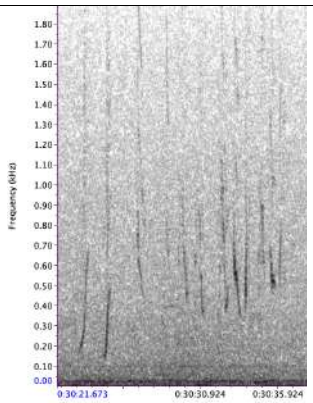

ASSO

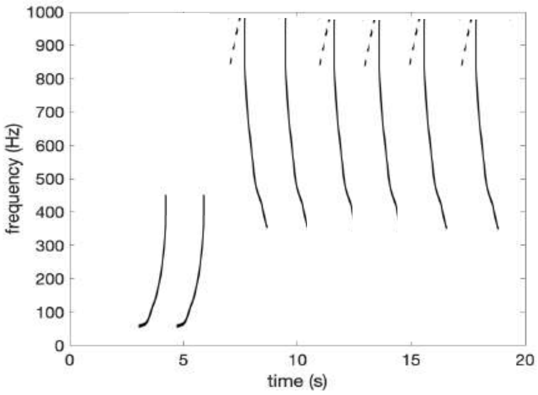

Type Ec

Combination of units CT4b, CT5b

Example of Ec24

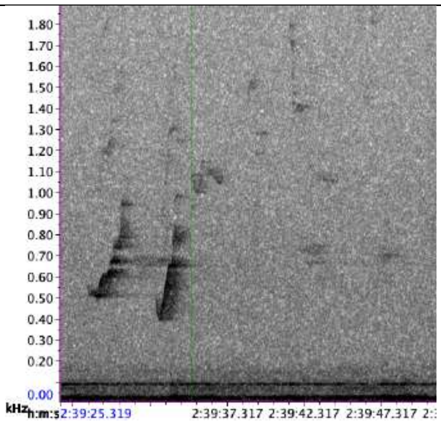

ASSO, B, E

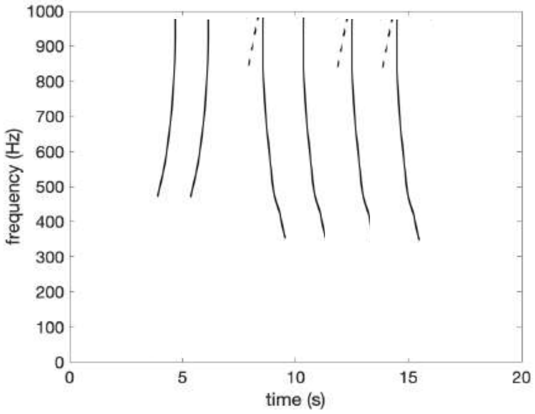

Type Ed

Combination of units CT4b, CT1

Example of Ed31	
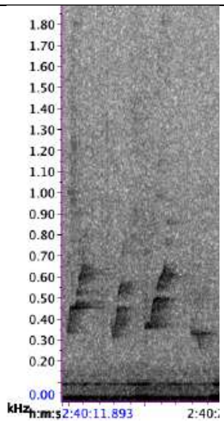	ASSO, E, B 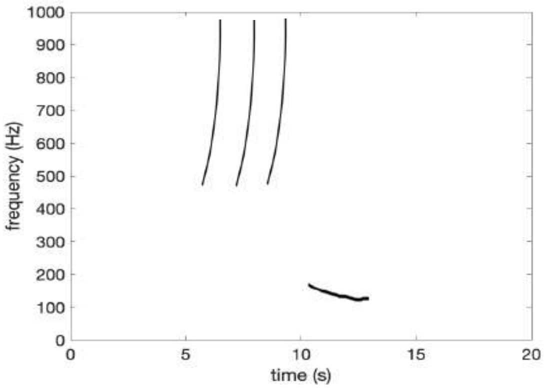

Type Ee

Combination of units CT4a, CT4b

Example of Ee11	
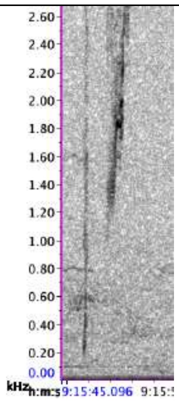	ASSO, B 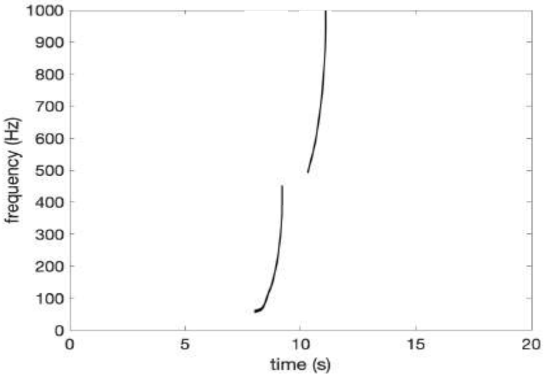

Type Ef

Combination of units CT4a, CT6

Example of Ef11	
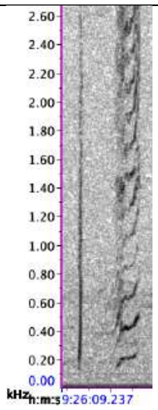	ASSO, B<br>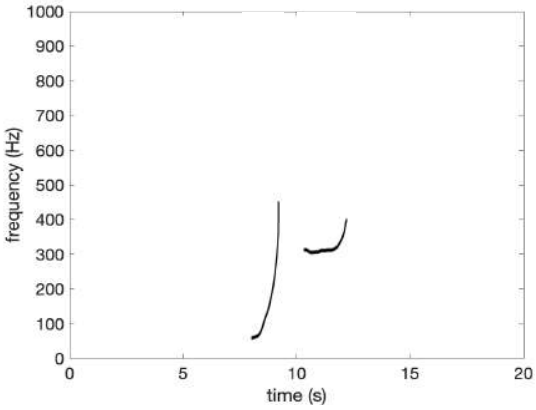

Type Eg

Combination of units CT4a, CT6, CT4b

Example of Eg111	
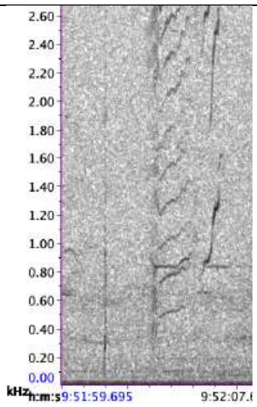	ASSO<br>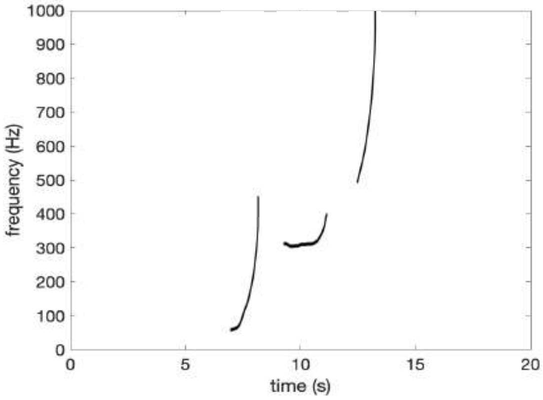

Type Eh

Combination of units CT4b, CT10

Example of Eh24

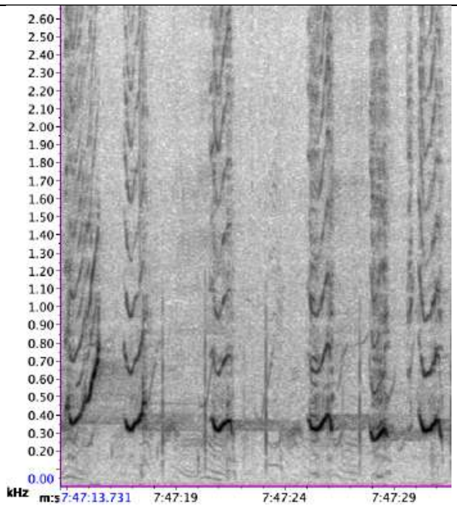

B

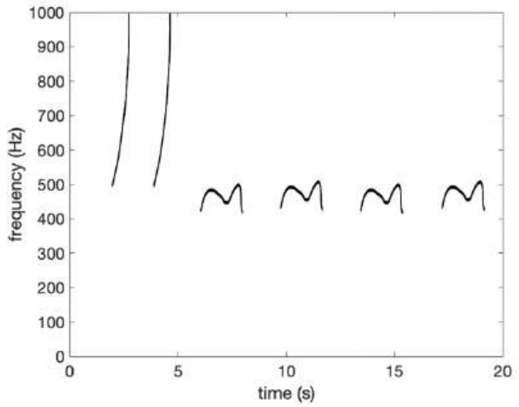

Type Fa

Combination of units CT18, CT5b

Example of Fa16

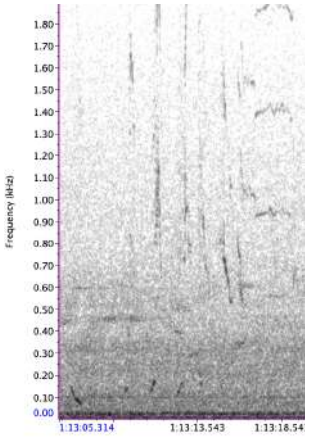

ASSO

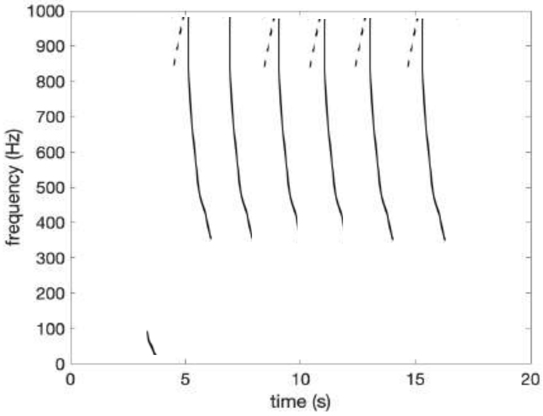

Type Fb

Combination of units CT18, CT1

<table>
<tr><th colspan="2">Example of Fb11</th></tr>
<tr><td>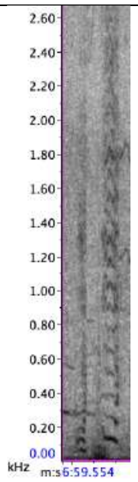
</td><td>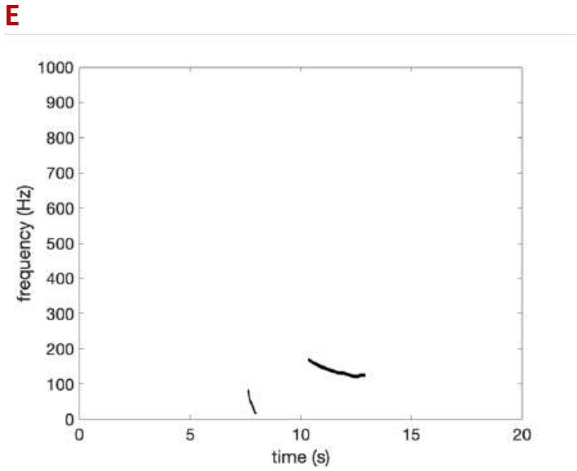
</td></tr>
</table>

Type Fc

Combination of units CT18, CT13a

<table>
<tr><th colspan="2">Example of Fc11</th></tr>
<tr><td>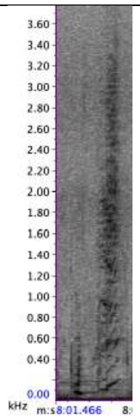
</td><td>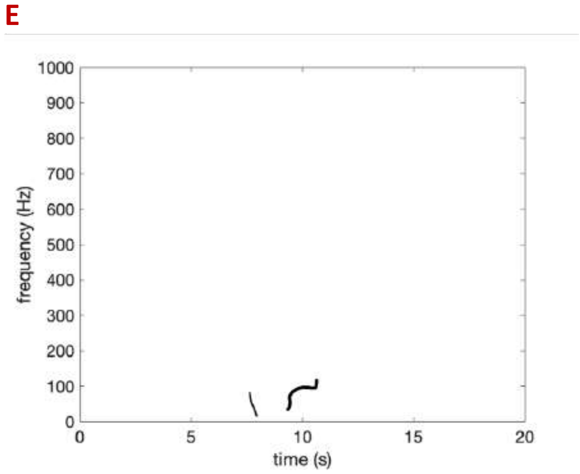
</td></tr>
</table>

Type Ga

Combination of units CT5a, CT19

Example of Ga11	
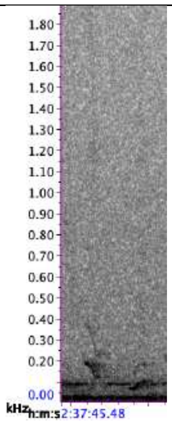	**ASSO, E** 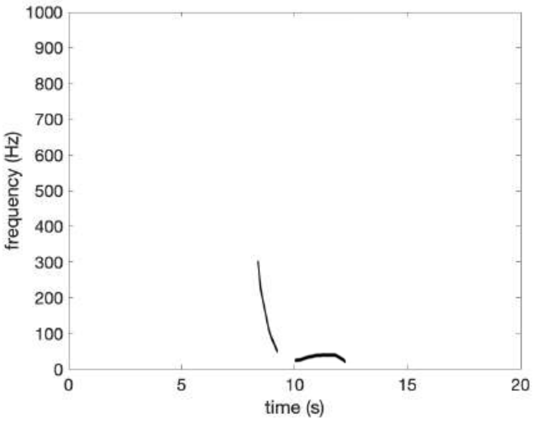

Type Gb

Combination of units CT5a, CT1

Example of Gb11	
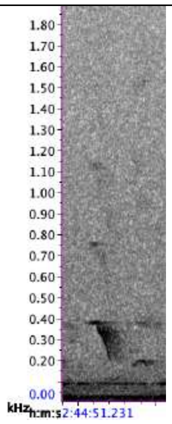	**ASSO, E, B** 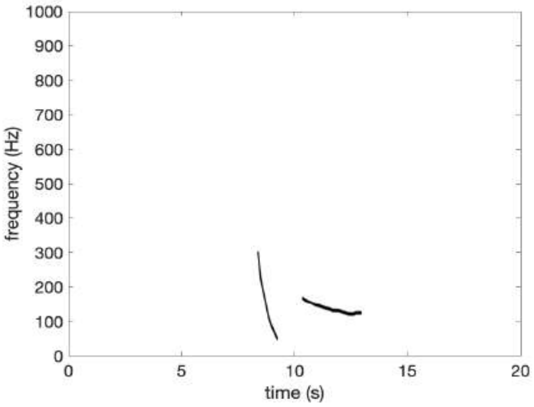

Type Gc

Combination of units CT5a, CT4a

Example of Gc11	
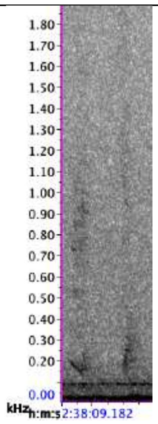	ASSO, B 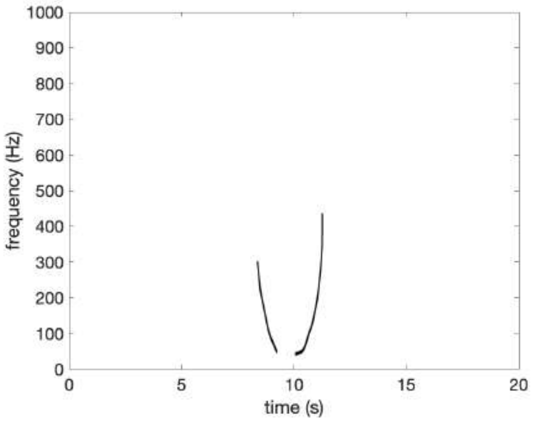

Type Gd

Combination of units CT5b, CT5a

Example of Gd12	
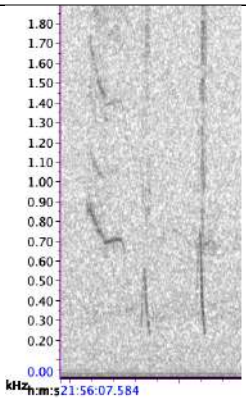	ASSO, A 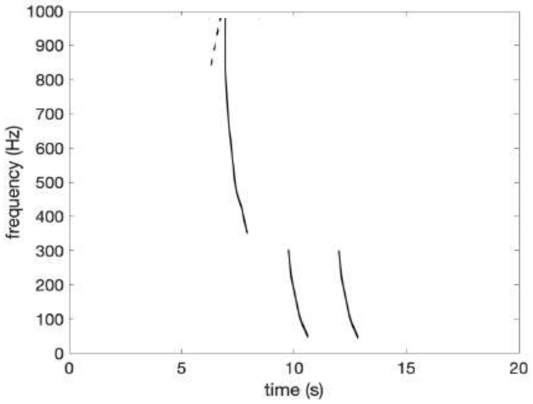

Type Ge

Combination of units CT5b, CT8

Example of Ge111	
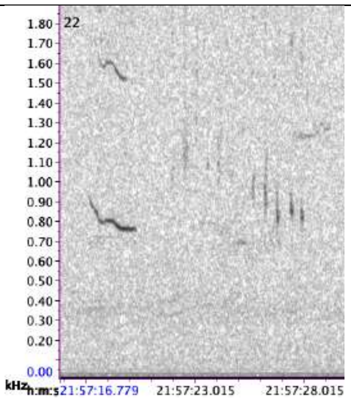	ASSO, A 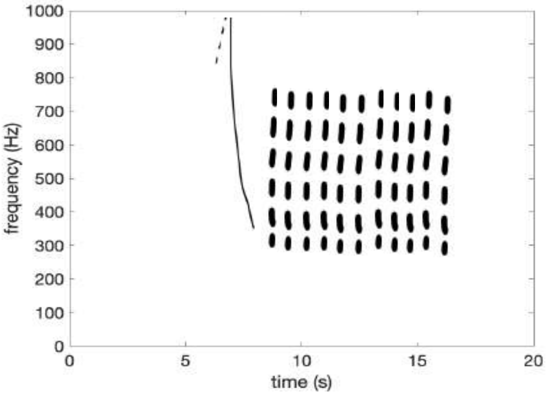

Type Gf

Combination of units CT5b, CT5a, CT8

Example of Gf1113	
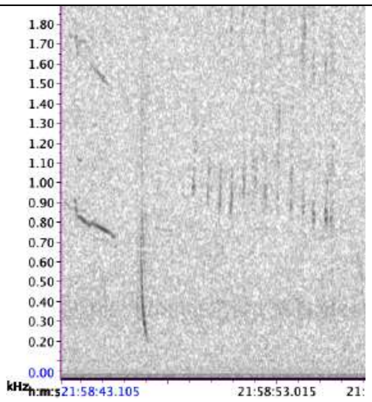	ASSO, A 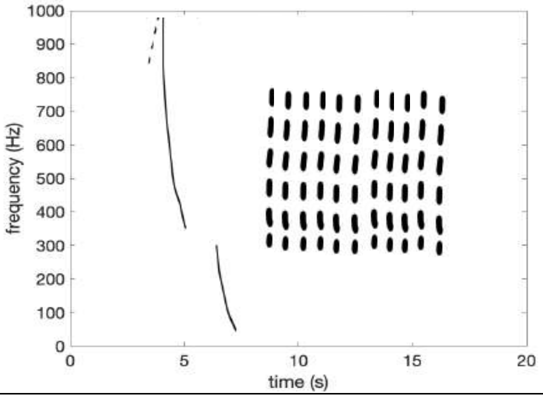

Type Gg

Combination of units CT5b/a

Example of Gg4

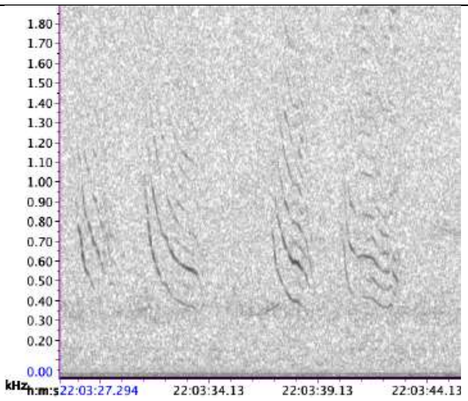

ASSO, A

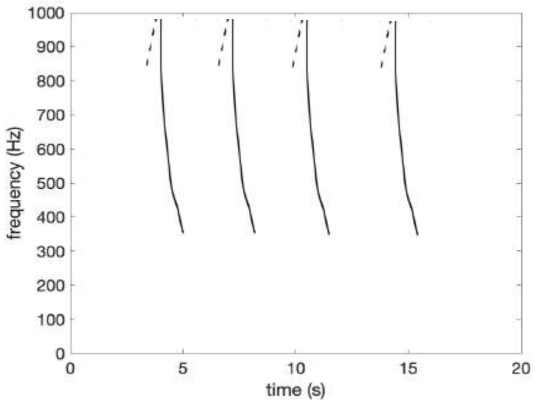

Type Gh

Combination of units CT5b, CT4a

Example of Gh27

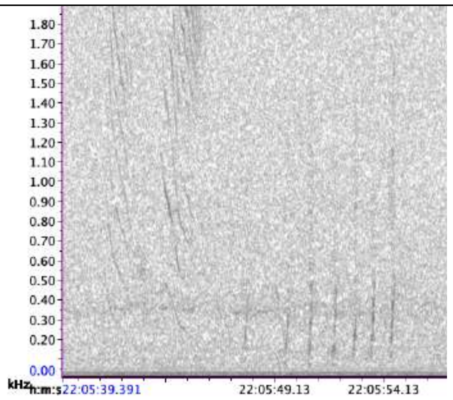

ASSO, A

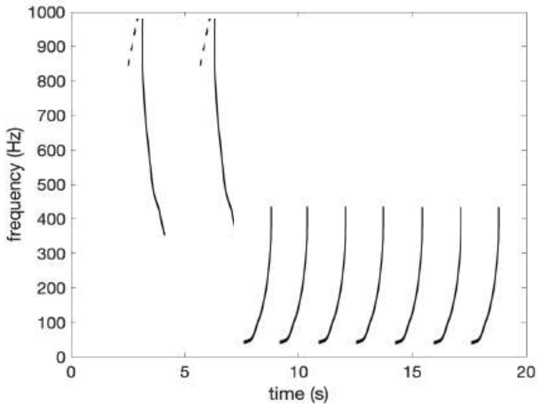

Type Gi

Combination of units CT5b, CT12

Example of Gi12	
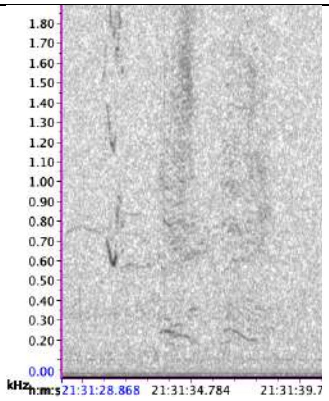	**ASSO** 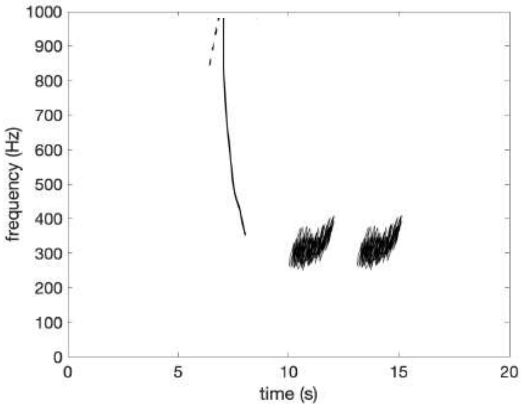

Type Gj

Combination of units CT5a, CT16, CT18

Example of Gj111	
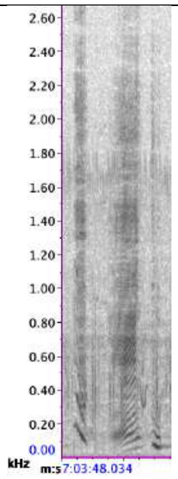	**B** 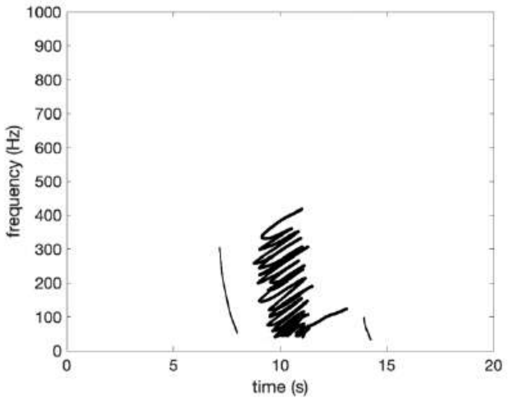

Type Gk

Combination of units CT5b, CT4b

<table>
<tr><th colspan="2">Example of Gk12</th></tr>
<tr><td>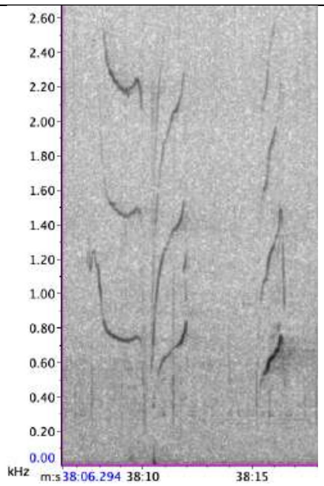
</td><td>B
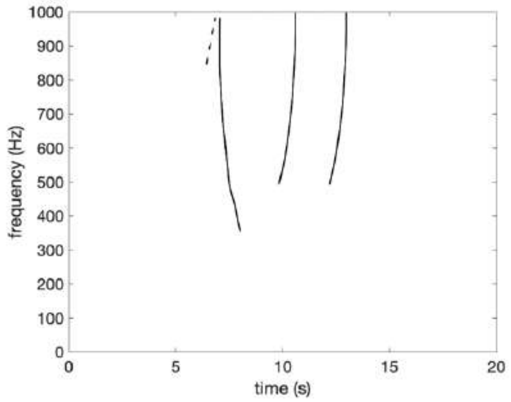
</td></tr>
</table>

Type Gl

Combination of units CT5a, CT10

<table>
<tr><th colspan="2">Example of Gl41</th></tr>
<tr><td>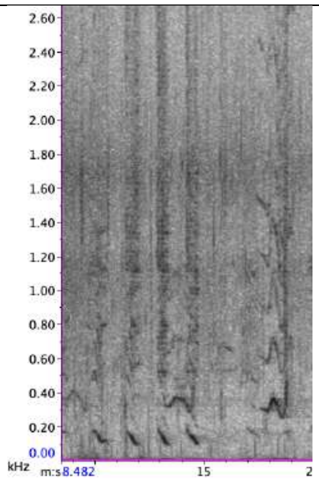
</td><td>E, B
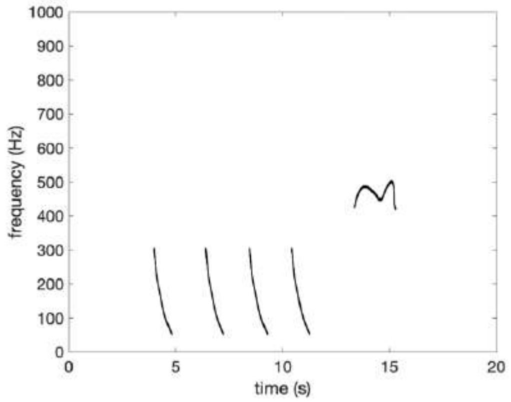
</td></tr>
</table>

Type Gm

Combination of units CT5a, CT1, CT15

<table>
<tr><th colspan="2">Example of Gm111</th></tr>
<tr><td>
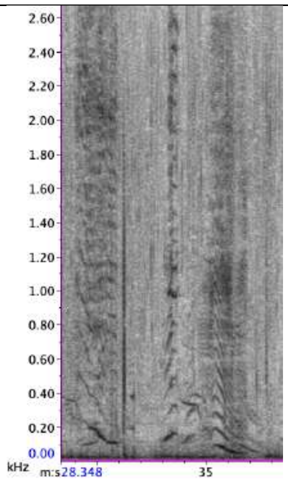

</td><td>
B

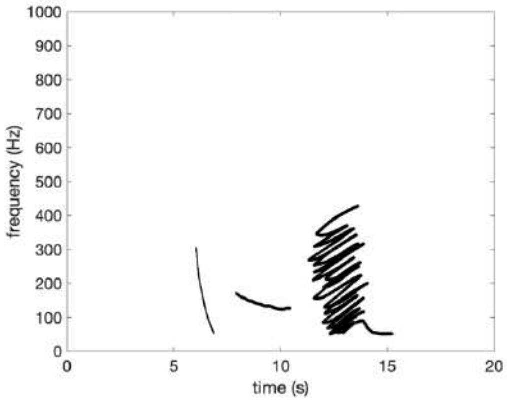

</td></tr>
</table>

Type Ha

Combination of units CT19, CT4a

<table>
<tr><th colspan="2">Example of Ha11</th></tr>
<tr><td>
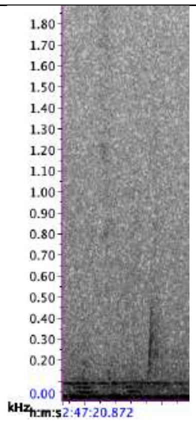

</td><td>
ASSO, E

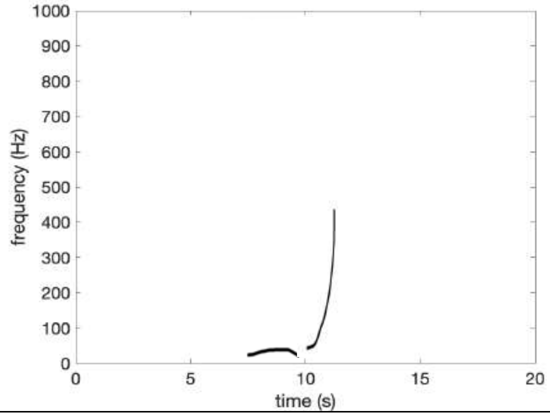

</td></tr>
</table>

Type Ia

Combination of units CT10

Example of Ia5

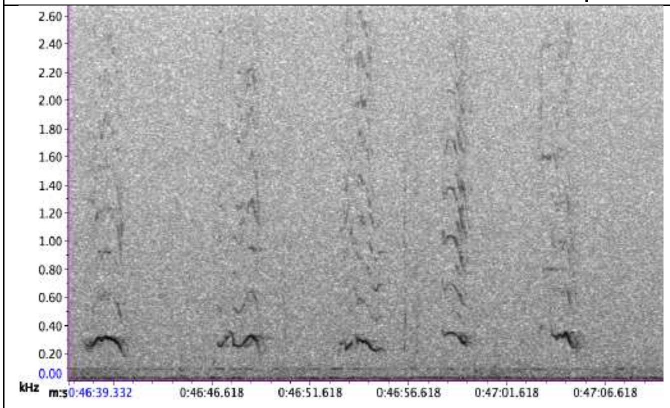

ASSO, E, B

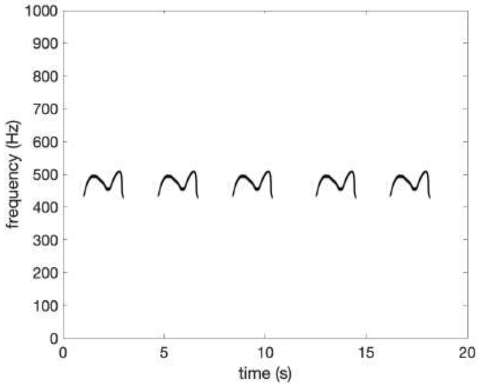

Type Ib

Combination of units CT10, CT8

Example of Ib12

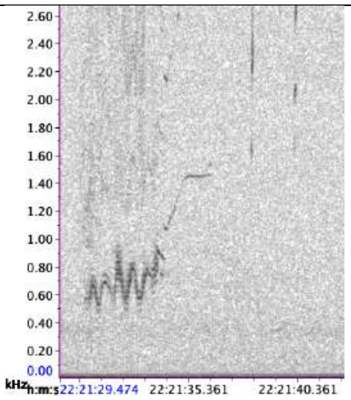

ASSO, A

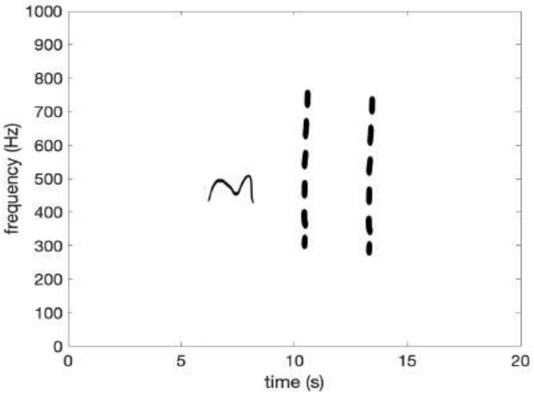

Type Ic

Combination of units CT10, CT13c

Example of Ic25

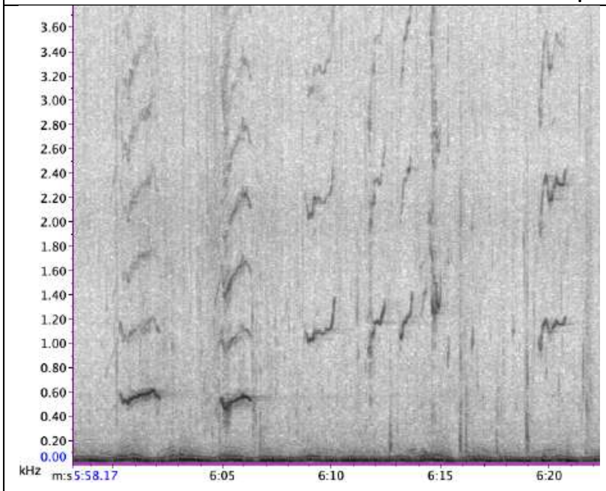

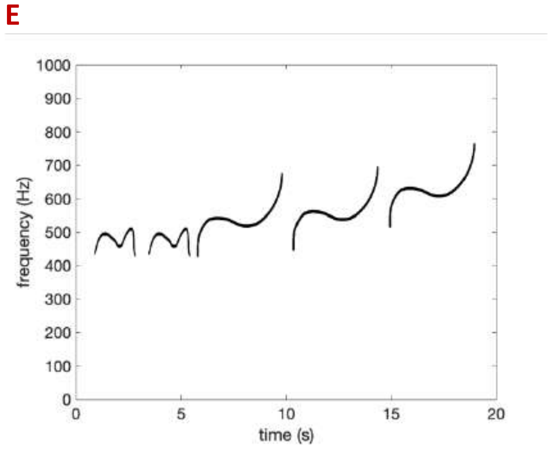

Type Id

Combination of units CT10, CT5b

Example of Id11

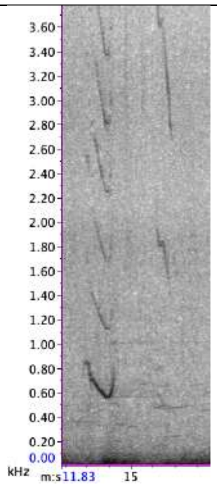

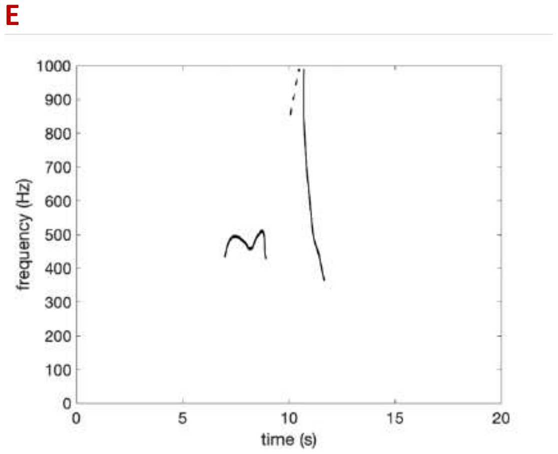

Type Ie

Combination of units CT10, CT1

Example of Ie12

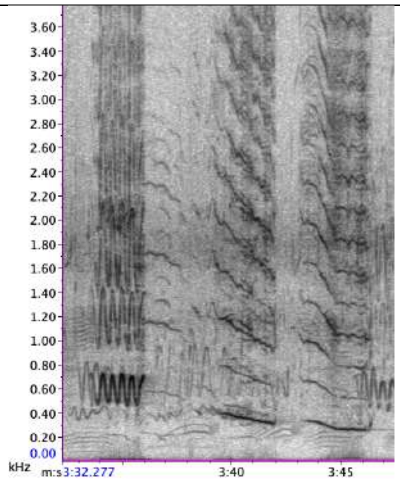

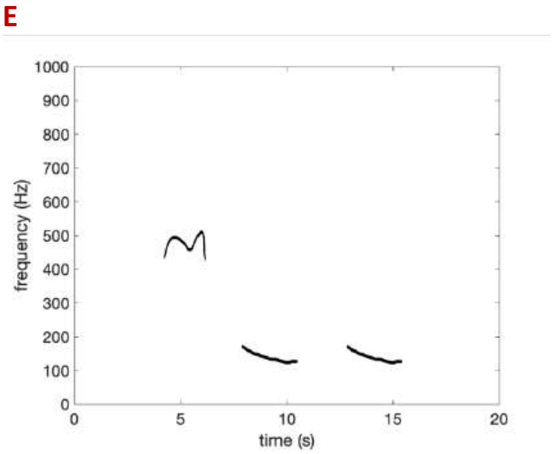

Type If

Combination of units CT10, CT8, CT1

Example of If161

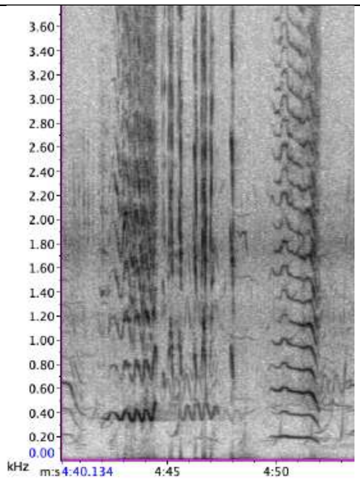

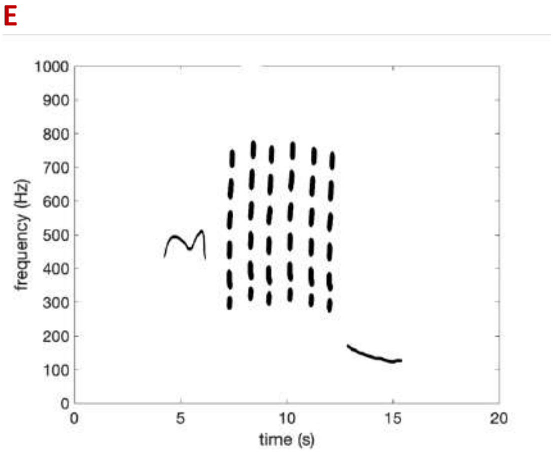

Type Ig

Combination of units CT10, CT19

Example of Ig13	
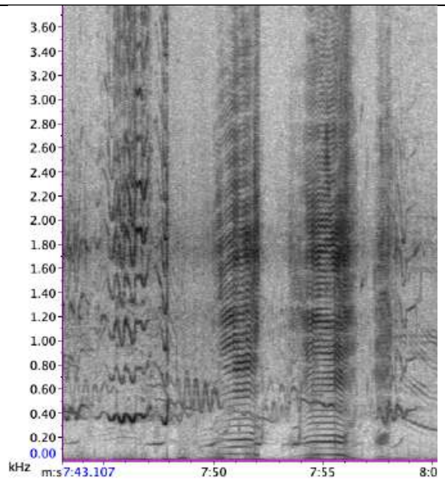	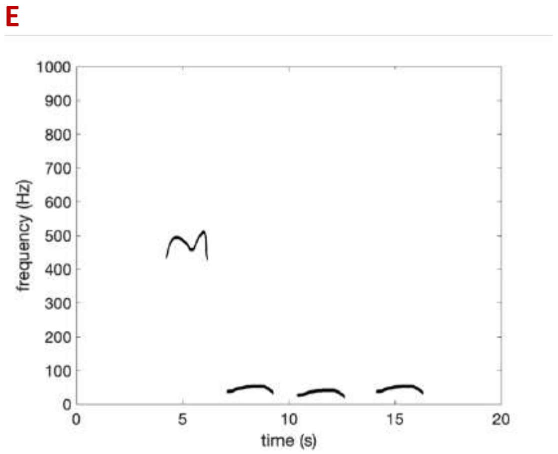

Type Ih

Combination of units CT10, CT1, CT13a

Example of Ih132	
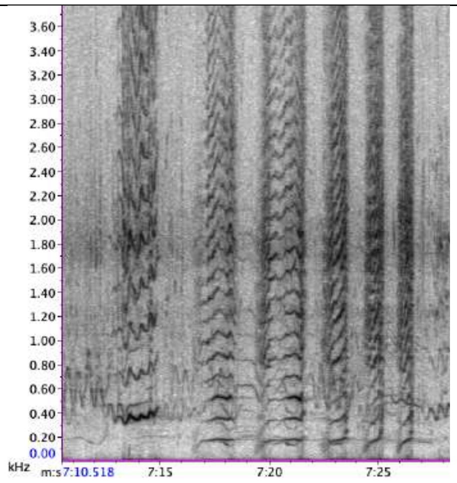	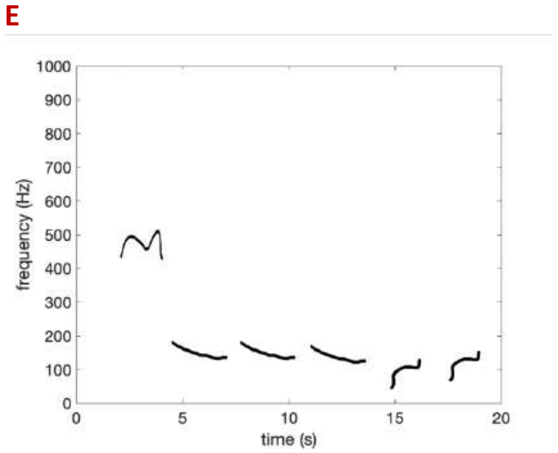

Type Ii

Combination of units CT10, CT13b, CT7

Example of Ii11312	
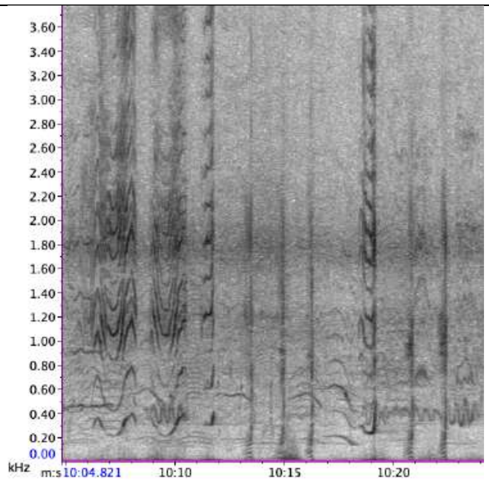	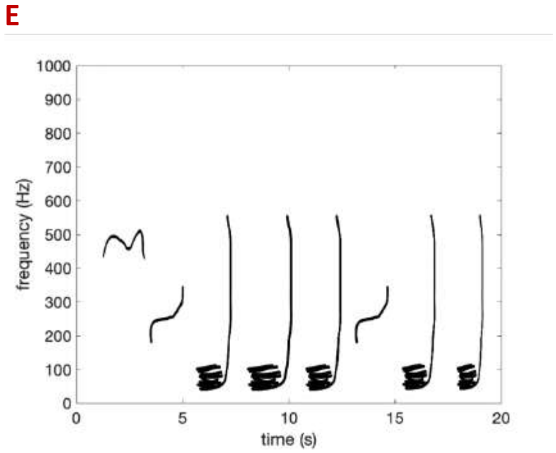

Type Ij

Combination of units CT10, CT4a

Example of Ij16	
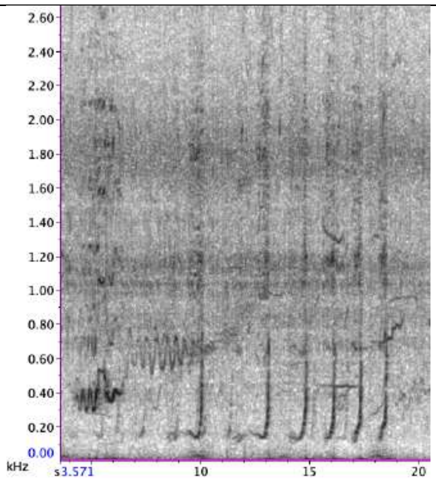	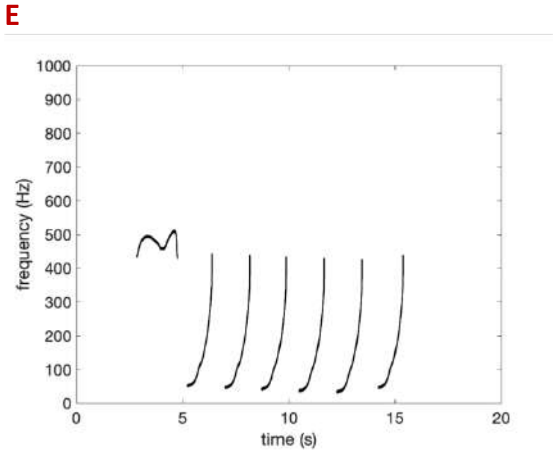

Type Ik

Combination of units CT10, CT19, CT4a

Example of Ik11111

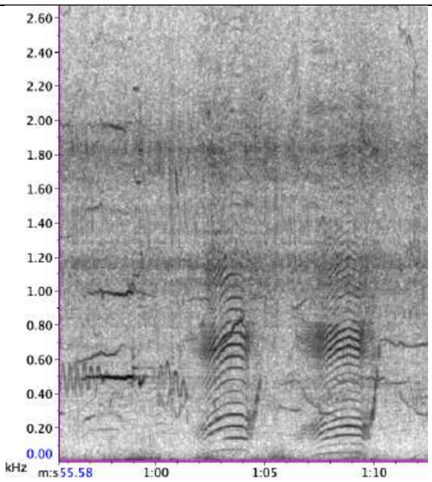

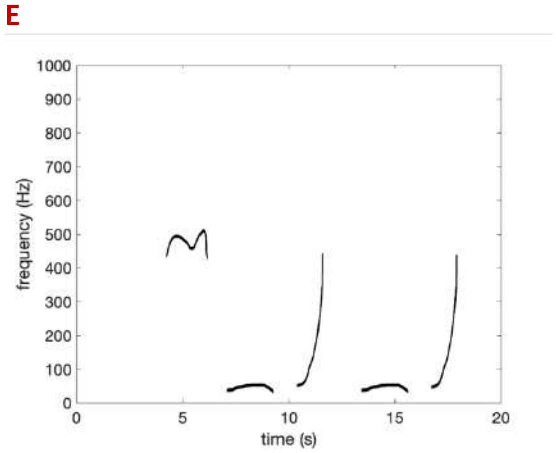

Type Il

Combination of units CT10, CT4b

Example of Il13

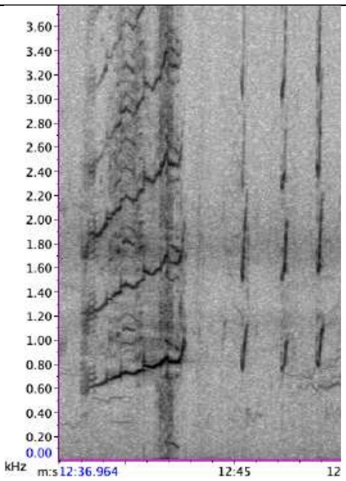

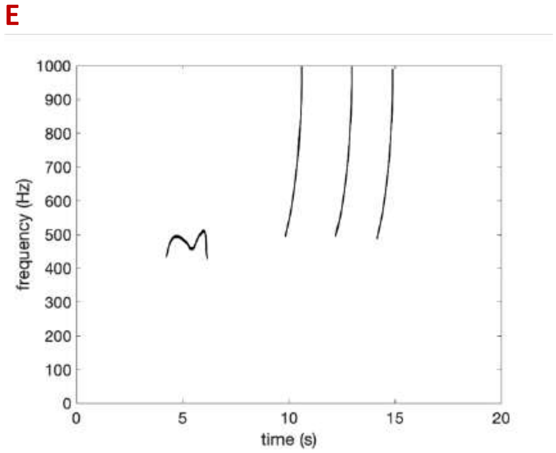

Type Ja

Combination of units CT7, CT4a

Example of Ja11	
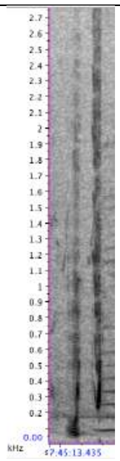	**B, E**<br>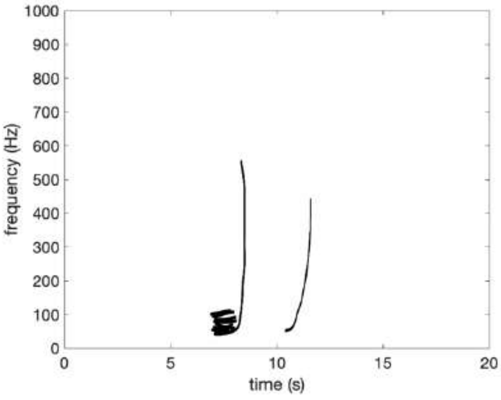

Type Jb

Combination of units CT7, CT12

Example of Jb11	
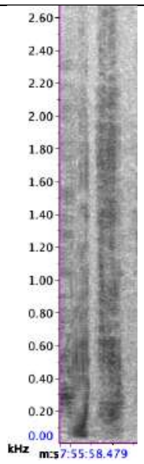	**B, E**<br>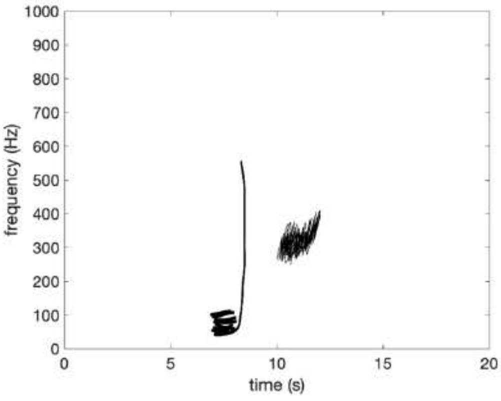

Type Ka

Combination of units CT16, CT18

Example of Ka11	
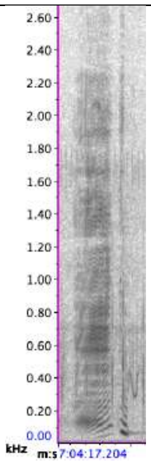	**B** 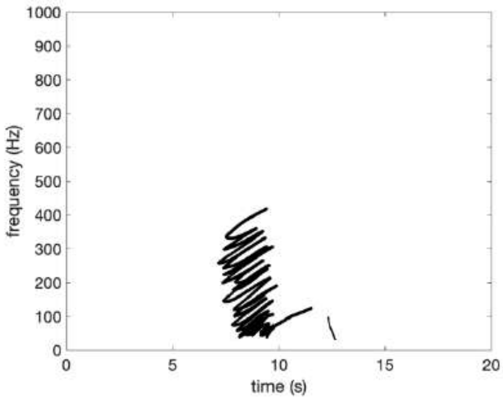

Type Kb

Combination of units CT16, CT12

Example of Kb11	
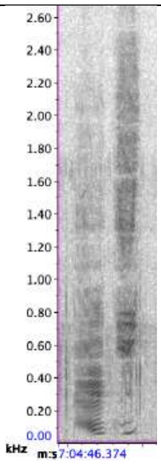	**B** 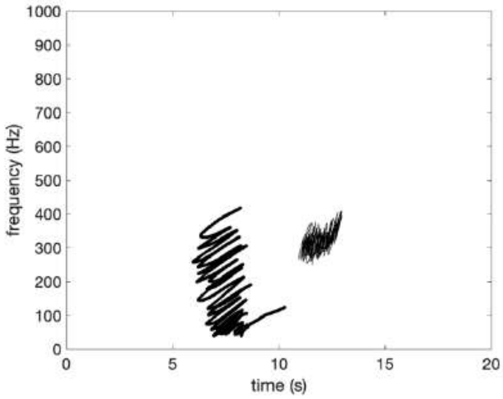

Type Kc

Combination of units CT16, CT10

Example of Kc11

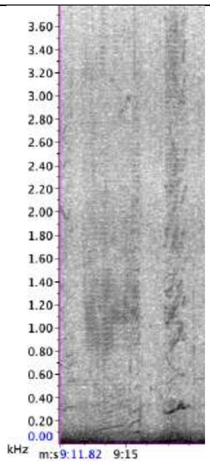

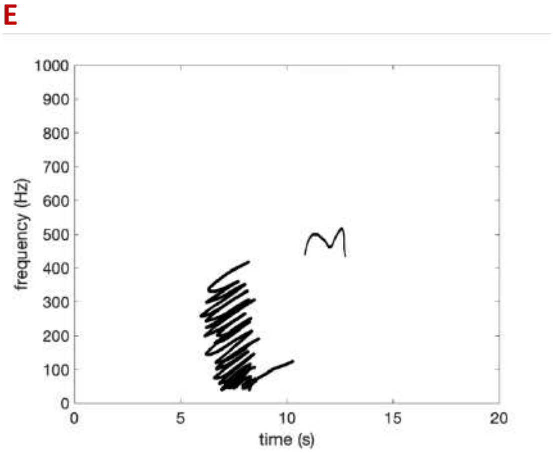

Type Kd

Combination of units CT16, CT13b

Example of Kd11

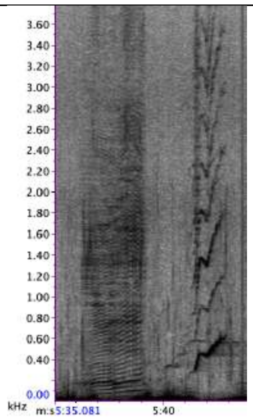

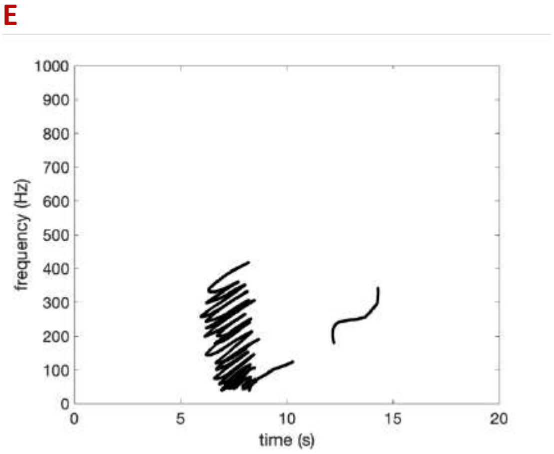

Type La

Combination of units CT13a, CT13b

<table>
<tr><th colspan="2">Example of La41</th></tr>
<tr><td>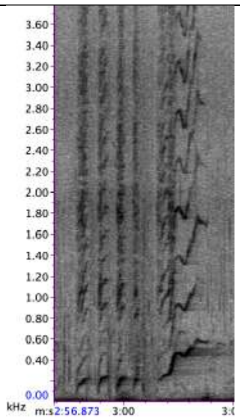
</td><td>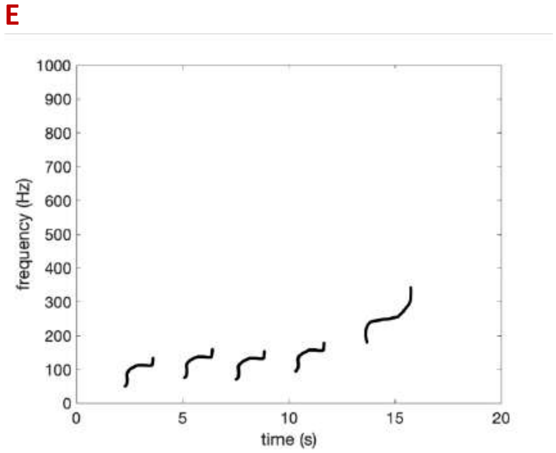
</td></tr>
</table>

Type Lb

Combination of units CT13b, CT10

<table>
<tr><th colspan="2">Example of Lb11</th></tr>
<tr><td>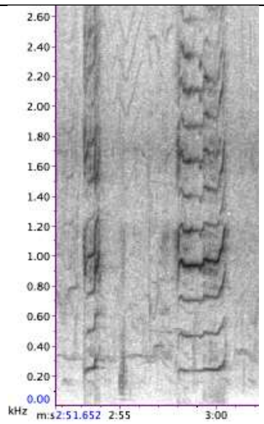
</td><td>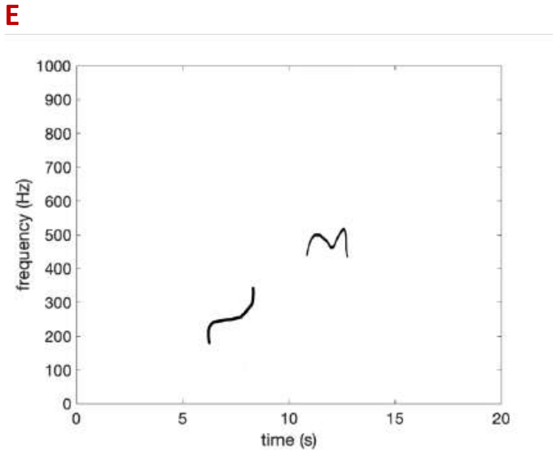
</td></tr>
</table>

Type Lc

Combination of units CT13a, CT19, CT4b

Example of Lc1112

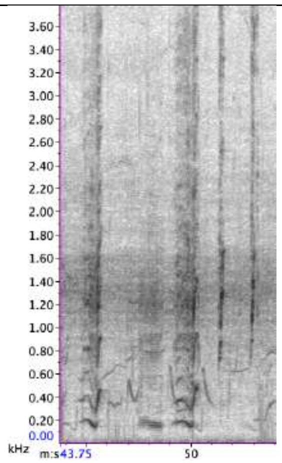

E

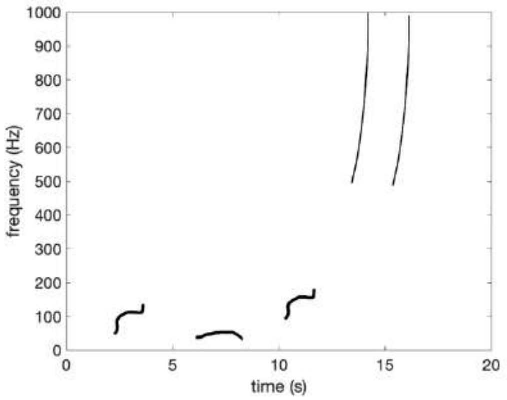

Type Ld

Combination of units CT13a, CT7, CT18

Example of Ld111111

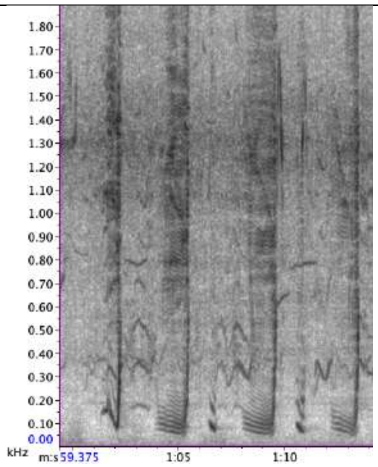

E

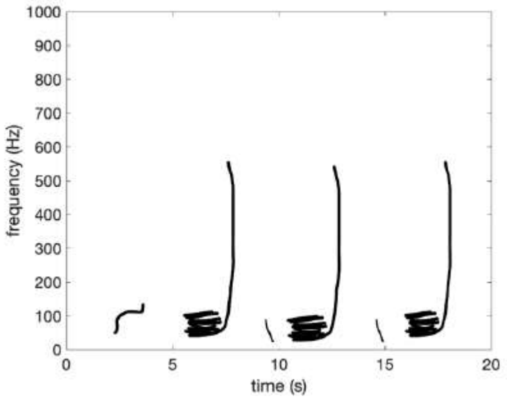

Type Ma

Combination of units CT17, CT4a

<table>
<tr><th colspan="2">Example of Ma11</th></tr>
<tr><td>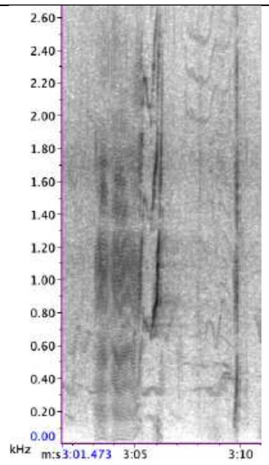
</td>
<td>E
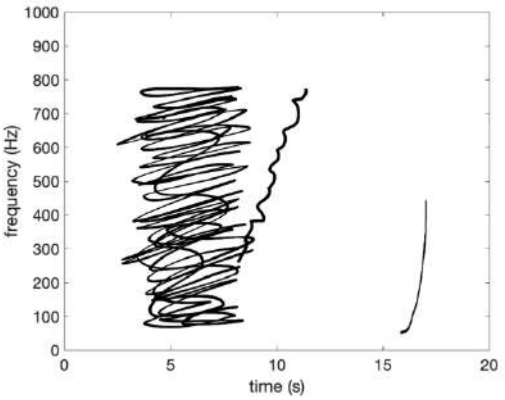
</td></tr>
</table>

Type Mb

Combination of units CT17, CT10, CT4a

<table>
<tr><th colspan="2">Example of Mb111</th></tr>
<tr><td>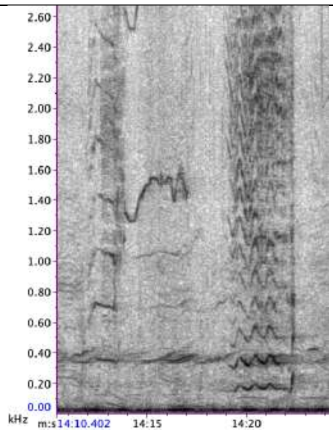
</td>
<td>B
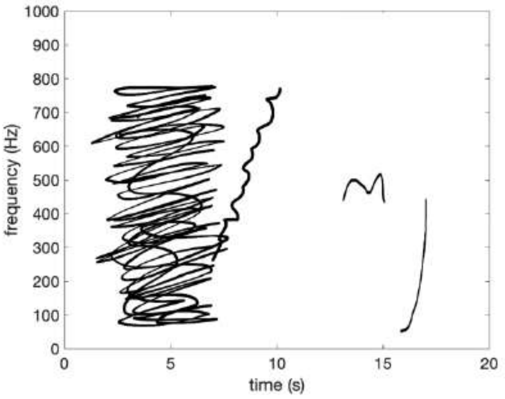
</td></tr>
</table>

Type Na

Combination of units CT15, CT10

Example of Na11	
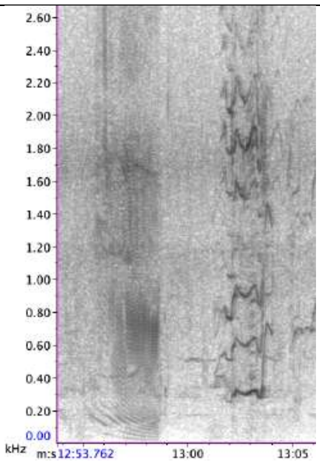	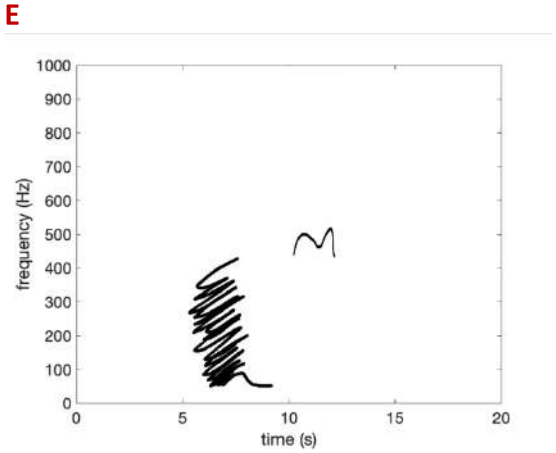
